# Harder than Metal: Challenging Antimicrobial Resistance with Metallo-β-lactamase Inhibitors

**DOI:** 10.1021/acs.jmedchem.5c00553

**Published:** 2025-05-30

**Authors:** Antonietta De Falco, Antonella Ilenia Alfano, Luigi Cutarella, Mattia Mori, Margherita Brindisi

**Affiliations:** † Department of Pharmacy (DoE 2023-2027), 9307 University of Naples Federico II, via D. Montesano 49, 80131 Naples, Italy; ‡ Department of Biotechnology, Chemistry, and Pharmacy, 9313 University of Siena, via Aldo Moro, 2, 53100 Siena, Italy

## Abstract

The spread of antimicrobial resistance (AMR) represents a major global health challenge, weakening the efficacy of antibiotics such as β-lactams, which are, nowadays, the most widely used drugs for treating bacterial infections. Among the different resistance mechanisms, the production of β-lactamases, particularly metallo-β-lactamases (MBLs), significantly compromises the activity of these antibiotics. Despite progress in developing serine-β-lactamase inhibitors (SBLi), no MBL inhibitors (MBLi) are currently available in clinical practice. This Perspective provides an outlook on AMR mechanisms, with a focus on the expression of MBL enzymes, and showcases the main classes of MBLi proposed to date, which mainly act through coordination of the zinc ion(s) populating the active site of the MBL class of enzymes. Furthermore, the Perspective describes current strategies aimed at overcoming the limited cellular permeability of MBLi, one of the major hurdles preventing their translation into clinical studies.

## Graphical abstract

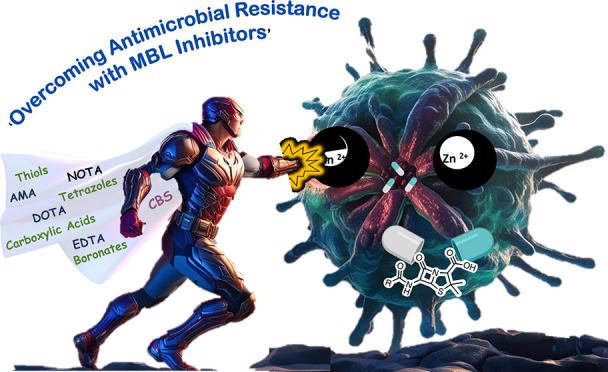

## Significance

- The term “antimicrobial resistance” refers to the ability of a bacterium to become insensitive or less sensitive to one or more antibiotics.
- The hydrolysis of the β-lactam ring, catalyzed by β-lactamase enzymes, represents one of the most important mechanisms for antimicrobial resistance.
- Metallo-β-lactamases (MBLs), featuring a catalytic site with one or two zinc ions, are expressed in several bacteria causing infections with a high mortality rate, since no metallo-β-lactamase inhibitors (MBLi) are currently available in the clinical practice.
- This Perspective showcases the main classes of MBLi proposed to date and describes the current strategies aimed at overcoming their limited cellular permeability.

## 1 Introduction

### 1.1 β-Lactam Antibiotics

β-Lactam antibiotics are nowadays the most widely employed drugs in clinical practice for the treatment of bacterial infections^1^ and are classified into four major classes: (i) penicillins, (ii) cephalosporins, (iii) carbapenems, and (iv) monobactams (general structures in Figure 1). These drugs possess β-lactam rings as a common feature in their chemical structure. The β-lactam system is a four membered cyclic amide ring highly reactive and susceptible to nucleophilic attack.^2^ The progenitor of this class is penicillin G, identified by Alexander Fleming in 1928 from *Penicillium notatum*’s metabolites and then isolated by Ernst Boris Chain and Lord Howard Florey in 1940.^3^ This discovery earned them the first Nobel Prize in Physiology or Medicine in 1945.^4^

**Figure 1:**
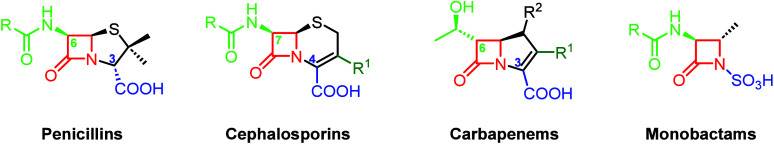
General structures of the four major classes of β-lactam antibiotics.

All β-lactam antibiotics are classified as bactericidal agents,^5^ since they behave as inhibitors of bacterial cell wall synthesis, essential for the bacteria’s normal growth and development.^6^ Their mechanism of action (Figure 2) is strongly related to the structural similarity between the β-lactam ring and the d-Ala-d-Ala dipeptide, a cell wall precursor of Gram-positive and Gram-negative microorganisms, involved in cross-linking the glycan chains in the peptidoglycan layer, thus conferring mechanical stability and rigidity to the cell wall.^7,8^ Due to this similarity, β-lactam antibiotics are able to interact with the catalytic site of the transpeptidase enzymes, generating a complex that, for steric reasons, inhibits the interaction between the enzyme and its substrate, the d-Ala-d-Ala dipeptide. Consequently, the cross-linking reaction involving the glycan chains in the peptidoglycan layer cannot occur.^9^ As a result, bacterial cell walls are characterized by weaker bonds, making them more prone to lysis and death.^5^

**Figure 2:**
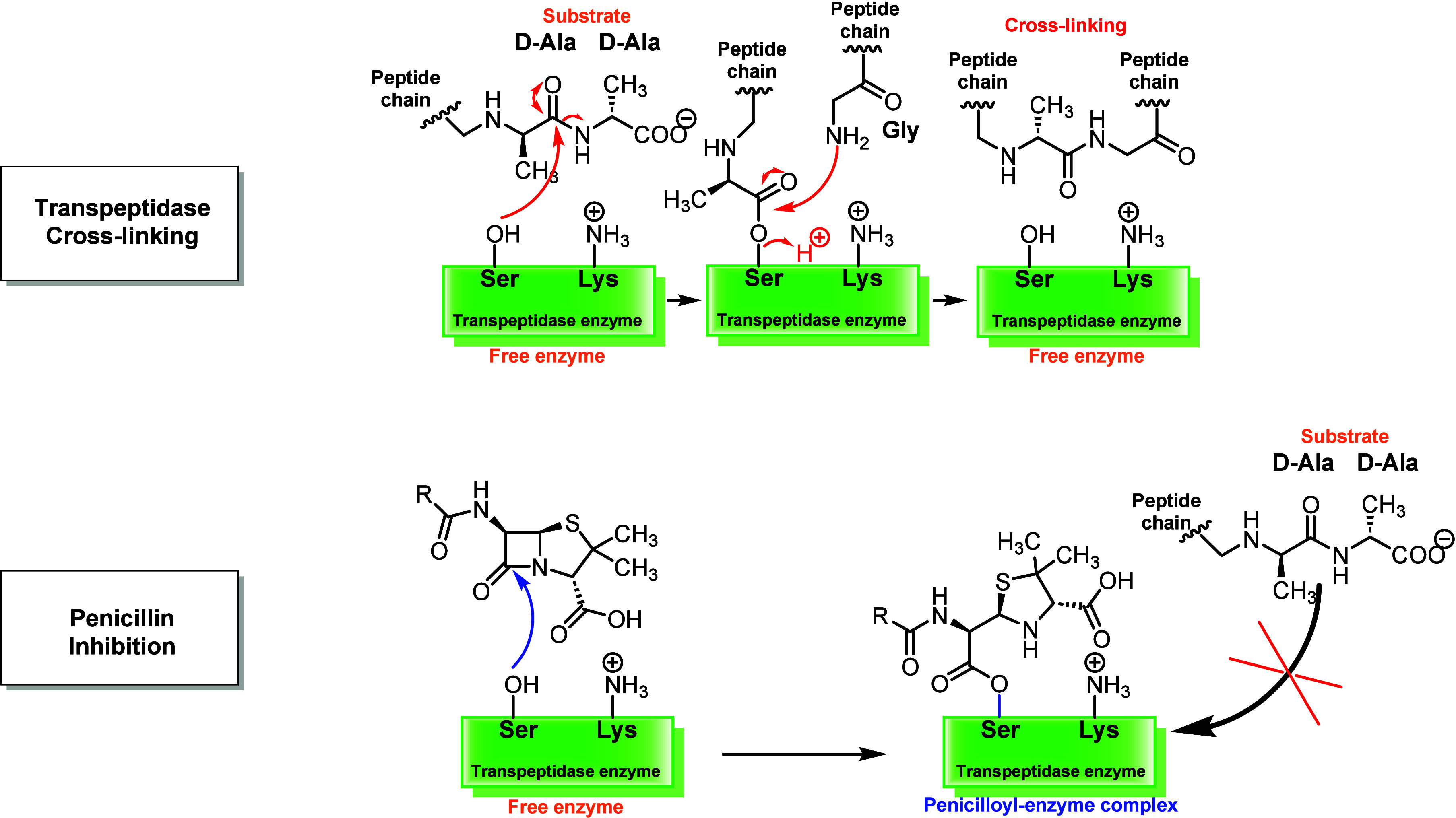
Mechanism of transpeptidase cross-linking and inhibition by penicillins.

### 1.2 Antimicrobial Resistance (AMR): Causes and Numbers

Although β-lactam antibiotics are the most used antibiotics worldwide, their efficacy is being seriously threatened due to the rise and spread of antimicrobial resistance (AMR). This term refers to the ability of a bacterium to become insensitive or less sensitive to one or more antibiotics (multidrug resistance, MDR), even in concentration generally sufficient to inhibit its multiplication or capable of killing it.^10^ AMR represents nowadays a major health problem worldwide, since current estimates predict it as one of the main causes of death in 2050 (Figure 3).^11,12^

**Figure 3:**
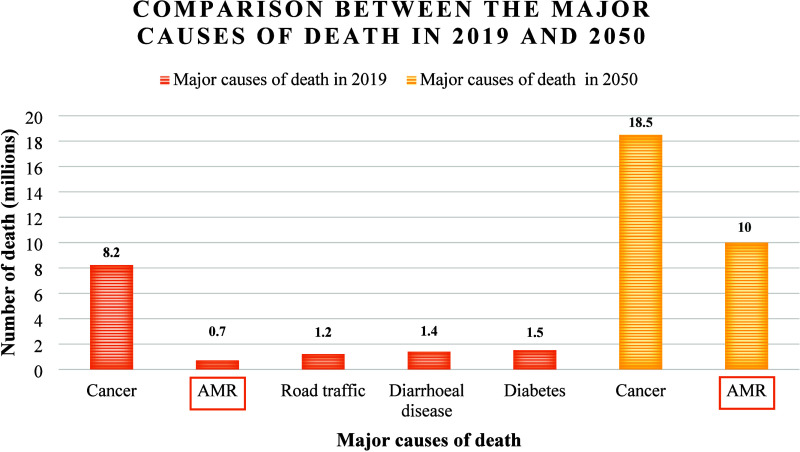
Rates of the major causes of death in 2019 and the estimate at 2050, with a highlight on AMR (Antimicrobial resistance).

Many factors contribute to antimicrobial resistance (Figure 4), although the main driving cause seems to be related to the overuse of antibiotics in the human, agricultural, and livestock sectors.^13^ In human medicine, overuse is often linked to improper dosage and risky self-medication.^14^ In livestock breeding, antibiotics are administered to animals to promote their growth and prevent pathologies, with two main consequences: the first is that the 75% of them are not absorbed and instead are eliminated from the body via urine and feces, which are widely used to fertilize agricultural soil, increasing the dissemination of resistance genes; the second is that the use of numerous antibiotics at subtherapeutic doses and for long periods has favored the fixation of resistance genes.^15,16^ Antibiotics are widely overused also in agriculture, mostly to prevent and cure various diseases in crops.^17^ Moreover, the COVID-19 pandemic has increased antibiotic resistance, since COVID-19 patients were heavily treated with broad-spectrum antibiotics, including extended-spectrum cephalosporins and carbapenems.^18^

**Figure 4:**
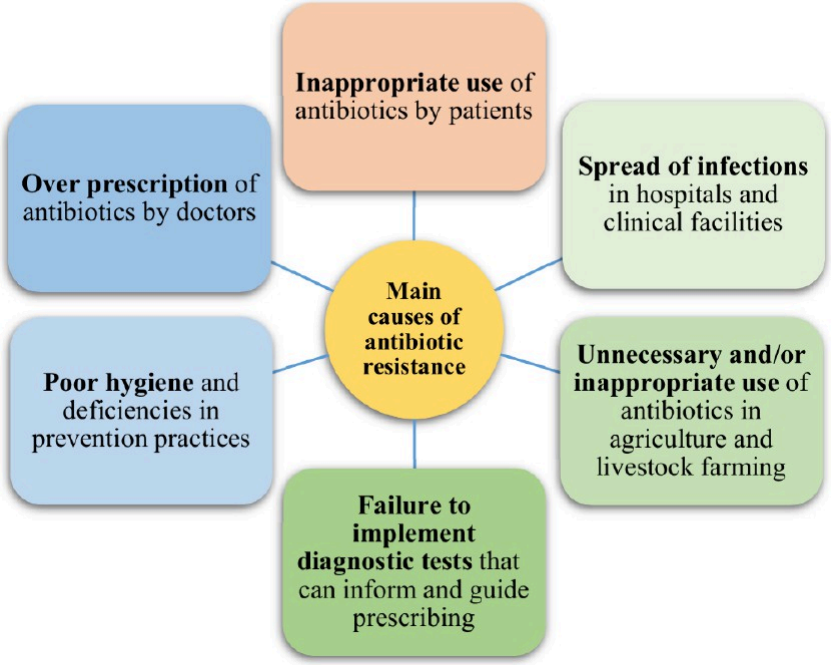
Main causes of antimicrobial resistance.

In particular, the most important and threatening pathogens are the well-known antimicrobial-resistant ESKAPE (*Enterococcus faecium, Staphylococcus aureus, Klebsiella pneumoniae, Acinetobacter baumannii, Pseudomonas aeruginosa, and Enterobacter species*) pathogens,^19^ that the World Health Organization (WHO), in February 2017, classified as “priority status” among the microorganisms for which new antimicrobial development is urgently needed.^20,21^ These pathogens, mainly through genetic mutation and the acquisition of mobile genetic elements (MGEs),^22^ have developed resistance mechanisms against a wide variety of drugs, including β-lactams, β-lactam−β-lactamase inhibitor combinations, and antibiotics that are the last line of defense, among which carbapenems, last-resort drugs, stand out.^19^

### 1.3 AMR Mechanisms

Antimicrobial resistance can be natural, intrinsic or adaptive, or acquired. The natural intrinsic resistance is always expressed and shared within a bacterial species. In the natural adaptive resistance, the genes are expressed only to resistance levels after exposure to an antibiotic. The acquired resistance is characterized by the acquisition, from other microorganisms, of genetic material conferring resistance.^23^ Antimicrobial resistance mechanisms toward β-lactam antibiotics are clustered into four main types (Figure 5): (i) limited uptake of a drug, (ii) modification of the drug target, (iii) drug efflux, and (iv) drug inactivation.^24^

**Figure 5:**
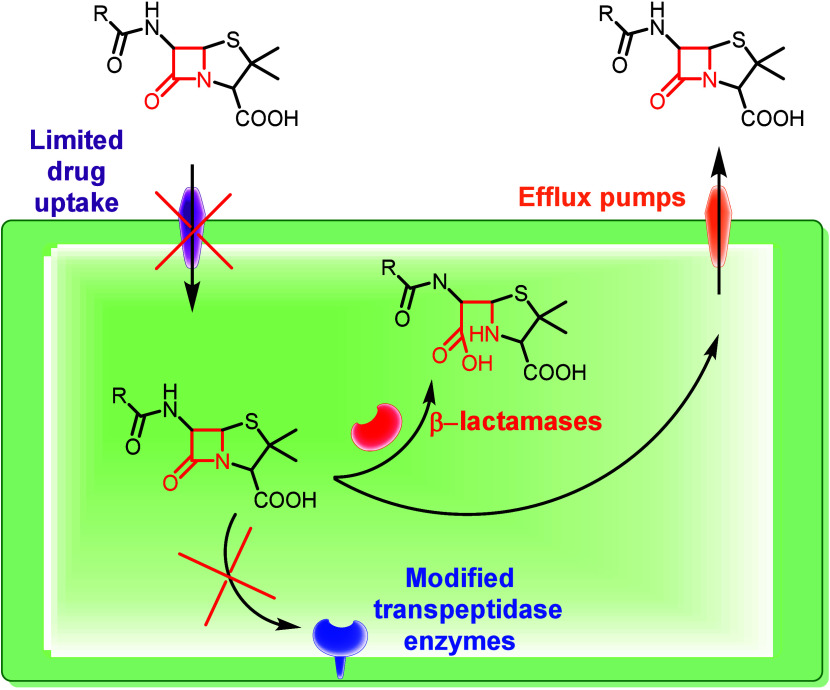
Main mechanisms of antimicrobial resistance.

### 1) Limited Uptake of a Drug

Bacteria possess different mechanisms through which they can limit the uptake of antimicrobial agents.^23^ Porin channels are often used by bacteria with large outer membranes to allow for the uptake of the drugs. Therefore, porin changes – through a decrease in the number of porins present or mutations that change the selectivity of the porin channels – can limit the drug uptake.^25^ Even the formation of a biofilm, characterized by a thick and sticky biofilm matrix, protects bacteria from the attack by the host immune system and limiting the uptake of antimicrobial agents.^26^

### 2) Modification of Drug Targets

Different components in the bacterial cells are targets of antimicrobial agents. Bacteria can enable resistance to drugs through modifications involving these targets. One mechanism of resistance related to β-lactam drugs, generally implemented by Gram-positive bacteria, is the alteration in the structure and/or number of the transpeptidase enzymes.^23^

### 3) Drug Efflux

Both Gram-positive and -negative bacteria possess chromosomally encoded genes for efflux pumps, which can be expressed constitutively, induced or overexpressed under certain environmental conditions;^23^ these efflux systems are capable of flushing out toxic compounds from the cell.^27,28^ Six types of efflux pumps have been described so far: (i) major facilitator superfamily, (ii) small multidrug resistance family, (iii) Resistance Nodulation Cell Divison family (RND), (iv) ATP binding cassette family (ABC), (iv) multidrug and toxic compound extrusion family (MATE), and vi) proteobacterial antimicrobial compound efflux (PACE).^29^

### 4) Drug Inactivation

Bacteria can inactivate drugs in two main ways: (i) by degradation of the drug, such as through β-lactamase enzymes, or (ii) by transfer of a chemical functionality to the drug, mainly acetyl, phosphoryl, and adenyl groups.^23^

Given the pressing threat posed by AMR, this Perspective focuses on the expression of β-lactamase enzymes, among the most relevant AMR mechanisms, with particular emphasis on metallo-β-lactamases (MBLs). A dedicated section then discusses the most clinically relevant MBLs (NDM, VIM, and IMP), focusing on their global spread and their role in resistant infections. The core of the Perspective provides a comprehensive overview of the main classes of MBL inhibitors (MBLi) proposed to date, highlighting the state-of-the-art in the field of MBLi, especially those displaying a clinical candidate profile. The subsequent section explores the major challenges affecting their *in vivo* efficacy, notably poor cellular permeability and limited periplasmic accumulation. The Perspective finally provides an overview of the most innovative strategies currently under investigation to address these limitations, such as modification of the physicochemical properties, Trojan horse conjugates, and nanoparticle-assisted delivery systems.

## 2 β-Lactamase Enzymes: General Features and Classification

Among the different antimicrobial resistance mechanisms, one of the most important – especially toward β-lactam based antibiotics – is the expression of the β-lactamase enzymes, which hydrolyze the β-lactam ring generating metabolites incapable of binding the transpeptidase enzymes.^1^ They were first identified in *Staphylococcus aureus* strains at the end of the 1940s, a few years after Penicillin G introduction into clinical practice.^30^ Following the Ambler classification, based on structural information, sequence similarity, and catalytic mechanism of action, β-lactamases are classified into four main classes: Ambler classes A-D (Figure 6, Table 1). Ambler classes A, C, and D are also known as serine β-lactamases (SBLs) as they possess a catalytic serine residue, responsible for the nucleophilic attack to the β-lactam ring and for its inactivation. Ambler class B contains instead MBLs, featuring a catalytic site in which one zinc ion (subclass B2) or two zinc ions (subclasses B1 and B3) are responsible for β-lactam ring hydrolysis.^31^ These MBLs exhibit a wide spectrum of action as they catalyze the hydrolysis of almost all β-lactam antibiotics, such as penicillins, cephalosporins, and carbapenems, except for monobactams.^31,32^ Among the most representative enzymes of each subclass there are (i) IMP (Imipenemase), VIM (Verona integron-encoded metallo-β-lactamase), and NDM (New Delhi metallo-β-lactamase) for the B1 subclass; (ii) CphA (a carbapenemase hydrolyzing enzyme from *Aeromonas hydrophila*) for the B2 subclass; and (iii) L1 (a labile enzyme from *Stenotrophomonas maltophilia*) and BJP (from *Bradyrhizobium japonicum*) for the B3 subclass.^11,31^

**Figure 6:**
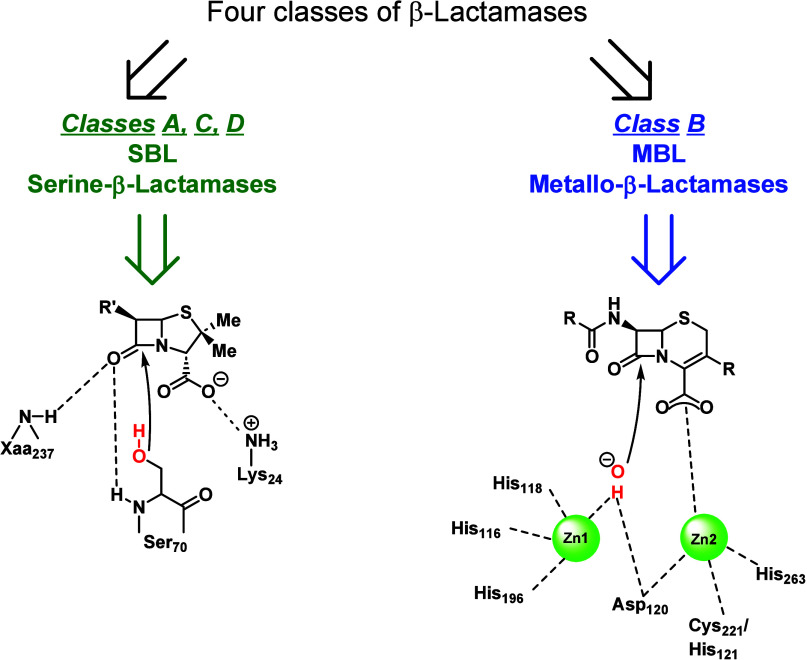
Ambler classification of β-lactamase enzymes and mechanism of hydrolysis of SBL and MBL.

**Table 1:** Ambler Classification, Representative β-Lactamase Isoforms, and Main Bacterial Strain Producers^31,33^

Class			Enzyme Name	Producers	β-Lactam substrates
SBLs	**Class A**		KPC	*K. pneumoniae, Serratiaspp*.	early generation penicillins
			TEM	*E. coli, K. pneumoniae, H. influenzae*	early generation cephalosporins
			GES	*K. pneumoniae, P. aeruginosa*	carbapenems
			CTX-M	*K. pneumoniae, E. coli*	aztreonam
			SHV	*K. pneumoniae, Enterobacterspp*.	
	**Class C**		ACT	*P. aeruginosa, A. baumannii, P. mirabilis*	penicillins
			DHA		early generation cephalosporins
			CMY		
			ADC		
	**Class D**		OXA-48	*Enterobacter spp.*	penicillins
			OXA-17	*P. aeruginosa, A. baumannii*	carbapenems
			OXA-23		
			OXA-24/40		
MBLs	**Class B**	**B1**	IMP	*S. marcescens, P. aeruginosa*	penicillins
			VIM	*P. aeruginosa, A. baumannii*	cephalosporins
			NDM	*K. pneumoniae*	carbapenems
		**B2**	CphA	*A. hydrophila*	carbapenems
		**B3**	L1	*S. maltophila*	penicillins cephalosporins carbapenems
			BJP	*B. japonicum*	

### 2.1 MBLs: Structure and Mechanism of Action

The zinc ion(s) in the active site of MBLs are essential for the catalytic mechanism of action, as they coordinate and activate a water molecule in the catalytic site, thus being responsible for the direct nucleophilic attack on the carbonyl group of the β-lactam ring and for its hydrolysis (Figure 7).^34^ MBLs are characterized by a very low sequence similarity among the three subclasses, which is mostly restricted to the conserved coordination environment surrounding the catalytic zinc ion(s). In contrast, when straying from metal binding sites, a significant degree of variation is observed among MBL isoforms. As confirmed by X-ray data, MBLs lack similarities with SBLs but all share (i) histidine, cysteine, and aspartate residues in their active sites and (ii) an αβ/βα sandwich scaffold with two central β-sheets, with the active site located in a shallow groove between the two facing β-sheets and five solvent-exposed α-helices.^31,35^

**Figure 7:**
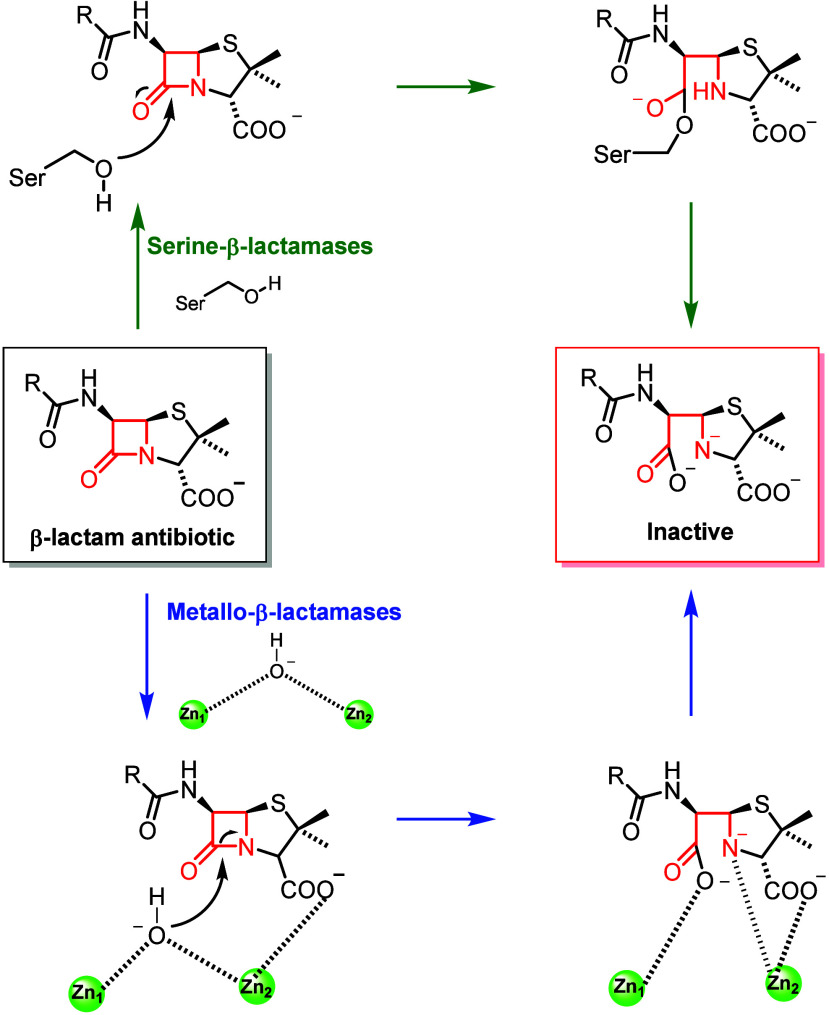
Catalytic mechanism of SBLs and MBLs.

## 3 Carbapenemases: Versatile β-Lactamases

Carbapenemases are β-lactamases that hydrolyze most penicillins, cephalosporins, and also carbapenems, as the name suggests. Some researchers have preferred the nomenclature “carbapenem-hydrolyzing enzymes” to the term “carbapenemases”, to highlight the fact that carbapenems are only a part of their broad spectrum of substrates.^36^ Carbapenemases belong to different classes of β-lactamases: some serine β-lactamases (e.g., KPC and OXA) can hydrolyze carbapenems, whereas all metallo-β-lactamases (e.g., NDM, IMP, and VIM) are carbapenemases. Since carbapenems are regarded as last-resort drugs,^37^ bacteria-producing carbapenemases – the well-known *“superbugs”*– are responsible for infections with a high mortality rate,^36,38^ and their appearance is mainly due to the spread of ESBLs (Extended-Spectrum β-Lactamases), that led to the improper and excessive use of these life-saving drugs.^39^ Nowadays, the number of carbapenemases is steadily increasing, and their genes on mobile genetic elements highly accelerate the spread of resistance through horizontal gene transfer across different species.^40^ Among the superbugs, one of the most significant contributing to the reduction of carbapenem efficacy in clinical practice is represented by the *carbapenem-resistant Enterobacteriaceae* (CRE), which has become a major public health problem worldwide since *E. coli* is one of the most important pathogens in humans. The main mechanism of carbapenem resistance in CRE is the acquisition of carbapenemase genes such as *bla*KPC, *bla*NDM, *bla*VIM, *bla*IMP, and *bla*OXA-48. CRE are distributed in 75 countries, mainly in the United States (17.49%), China (14.88%), and the United Kingdom (14.73%). In particular, NDM is the most predominant carbapenemase (52.15%), followed by OXA (30.09%) and KPC (14.72%).^41^

## 4 Clinically Relevant MBLs

The most clinically relevant MBLs belong to subclass B1. The β-lactamase genes (*bla* genes) encoding subclass B1 metallo-β-lactamases, such as IMP, VIM, and NDM, are generally plasmid-born, meaning that these enzymes can be transferred between bacterial strains via these mobile genetic elements. In particular, the IMP-type β-lactamases, identified in 1991 in Japan, remain the predominant MBLs in Southeast Asia and can be found among *P. aeruginosa*, *A. baumannii*, and different species of *Enterobacterales.* The VIM-type β-lactamases were discovered in 1997 in Italy and were the predominant MBLs in Europe until 2017; they are associated mostly with *P. aeruginosa* (VIM-2-like β-lactamases) and strains of *Enterobacterales* (VIM-1-like β-lactamases). The NDM-type β-lactamases, first identified in India in 2008, spread throughout the world, becoming the predominant MBLs in Europe. They have been reported in several families of *Enterobacterales* and in other Gram-negative bacteria, such as *Vibrio cholerae*, *Pseudomonas spp*, and *A. baumannii.* ^11^ The New Delhi Metallo-β-lactamase-1 (NDM-1) is nowadays considered as the most clinically relevant target for antibiotic resistance due to its worldwide prevalence.^42^ This is mainly ascribable to its cellular localization: NDM-1 is a lipoprotein anchored to the outer membrane in Gram-negative bacteria (Figure 8); conversely, all other MBLs are soluble periplasmic proteins.^43^ This cellular localization has two main consequences:

1. Increased resistance to the host immune system, since it prevents the proteolytic degradation of the apo-NDM-1 enzymes, generated after the activation of the host immune system and the release, at the sites of infection, of large amounts of the metal-chelating protein calprotectin (CP);^42^
2. Secretion of NDM-1 in outer membrane vesicles (OMVs), responsible for the gene transfer, and so for the spread of the resistance,^11^ and for the protection of nearby populations of carbapenem-susceptible bacteria, due to the potent carbapenemase activity of OMVs.^44^

**Figure 8:**
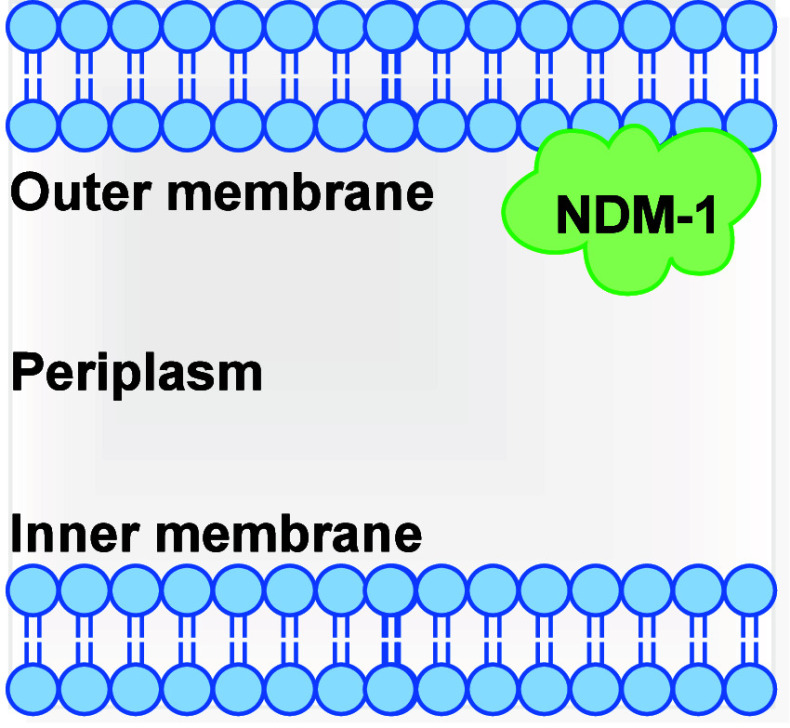
Schematic representation of cellular localization of NDM-1.

For these reasons, all the bacteria expressing NDM-1 are highlighted as Priority One by the WHO.^11^

## 5 Metallo Beta-Lactamase Inhibitors: Current Scenario

Currently, only SBL inhibitors (SBLi) are available in clinical practice (Table 2), while there are no clinically approved MBLi.^45^ The development of novel and effective MBLi is complicated by several factors: (i) MBLs’ active site is located in a shallow groove between the two facing β–sheets,^46^ in contrast with the deeper and more enclosed active site of SBLs; (ii) sequence similarity between subclasses is very low; and (iii) structures and the active site are often superimposable to those of other human metalloenzymes, causing important and undesirable off-target effects.^47^

**Table 2:** Summary Table of β-Lactam-SBLi Combinations Currently Available in Clinical Practice

	Year of FDA Approval	β-lactam Partner	Type of Inhibitor
**Clavulanic acid**	1984	Amoxicillin	I generation
**Sulbactam**	1987	Ampicillin	I generation
**Tazobactam**	1993	Piperacillin	I generation
**Tazobactam**	2014	Ceftolozane	I generation
**Avibactam**	2015	Ceftazidime	II generation
**Avibactam**	2024	Aztreonam	II generation
**Vaborbactam**	2017	Meropenem	III generation
**Relebactam**	2019	Imipenem and Cilastatin	II generation

The MBLi that have been evaluated so far mainly act through interaction with the zinc ions located in the active site of the MBLs, which are necessary for the structural stability and the mechanism of action of these enzymes.^31^ In particular, they mainly work through three different strategies (Figure 9): (i) metal ion stripping, (ii) displacement or locking of the Zn­(II)-complexed hydroxide/water molecule, and (iii) replacement of the metal cofactor with a metallodrug.^48^

**Figure 9:**
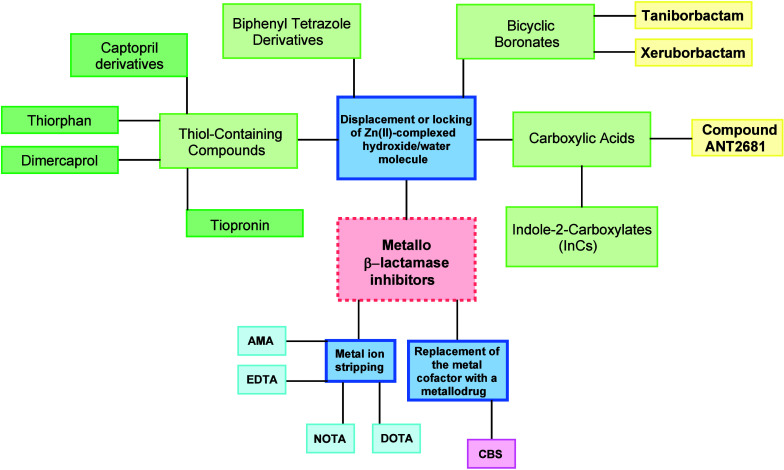
Summary chart of the most promising MBLi developed so far. Compounds in yellow are currently undergoing preclinical development (ANT2681) or clinical trials (taniborbactam and xeruborbactam). AMA: Aspergillomarasmine A; CBS: Colloidal bismuth subcitrate.

### 5.1 Metal Ion Stripping

The MBLi displaying their inhibitory activity via a metal ion stripping mechanism are strong chelating agents that strongly coordinate and then remove metal ions from the active site of MBL enzymes, thus inhibiting their catalytic mechanism of action. EDTA (**1**), Aspergillomarasmine A (AMA) (**2**), NOTA (**3**), and DOTA (**4**) (Figure 10) are the most representative compounds of this class. EDTA strongly binds the zinc ions of NDM-1, but its derivatives, except for Ca-EDTA, are not transferable into clinical practice, due to their nonspecific activity and cytotoxic profile.^49^ Ca-EDTA – the correspondent disodium calcium salt – was demonstrated by Yoshizumi et al. to be able to greatly potentiate the antibacterial activity of imipenem (IPM) and Meropenem (MEM) in different NDM-1 positive strains. In particular, when used at a final concentration of 32 μg/mL, the MICs (Minimum Inhibitory Concentration) of IPM and MEM were reduced from 64 μg/mL and 256 μg/mL, respectively, to 1 μg/mL in overexpressing NDM-1 *E. coli* TUM10701 strains.^50^ Aspergillomarasmine A (AMA) (**2**) – a fungal natural product identified by King and co-workers in 2014 – is able to restore MEM activity while maintaining nontoxic effects: a 95% survival rate has been achieved 5 days after mice infection with NDM-1 producing *K. pneumonia,* in contrast with the lower survival rate of Meropenem in monotherapy.^51^ 1,4,7-Triazacyclononane-1,4,7-triacetic acid (NOTA) (**3**)^52^ and 1,4,7,10-tetraazacyclododecane-1,4,7,10-tetraacetic acid (DOTA) (**4**)^53^ are metal-chelating agents able to restore the activity of carbapenems in MBL-producing bacterial infections more efficiently than Aspergillomarasmine A (MICs lower than those reported for AMA). Specifically, the Meropenem/NOTA combination (optimized concentration of NOTA: 4 mg/L) can inhibit MBLs’ activity better than the Meropenem/DOTA combination (optimized concentration of DOTA: at least eight times the NOTA’s one).^49,54^ Despite the promising results of these chelating agents inhibitors in both *in vitro* and i*n vivo* assays, the main issue preventing them from being used in clinical practice is their unspecificity, resulting in undesirable interactions with other metalloproteins (similar to MBLs) and divalent cations (such as Ca^2+^ or Mg^2+^) in the human body, thus leading to several off-target effects.^31^

**Figure 10:**
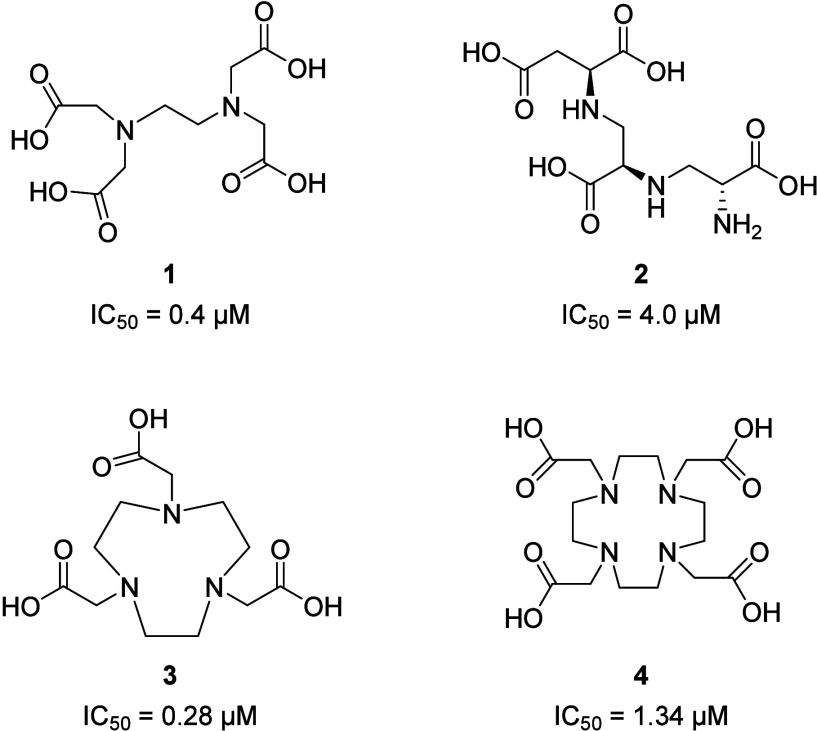
Structures of the representative strong chelating agents proposed as MBLi. Reported IC_50_ values are referred to NDM-1 isoform.

### 5.2 Displacement or Locking of the Zn­(II)-Complexed Hydroxide/Water Molecule

#### 5.2.1 Thiol-Containing Compounds

Thiol-containing compounds were found to be among the most promising categories. The progenitor of this class is (2*S*)-1-[(2*S*)-2-Methyl-3-sulfanylpropanoyl] pyrrolidine-2-carboxylic acid (commonly known as L-captopril) (**5;** Figure 11), a molecule belonging to the class of angiotensin-converting enzyme (ACE) inhibitors, used to treat hypertension and heart failure.^51^

**Figure 11:**
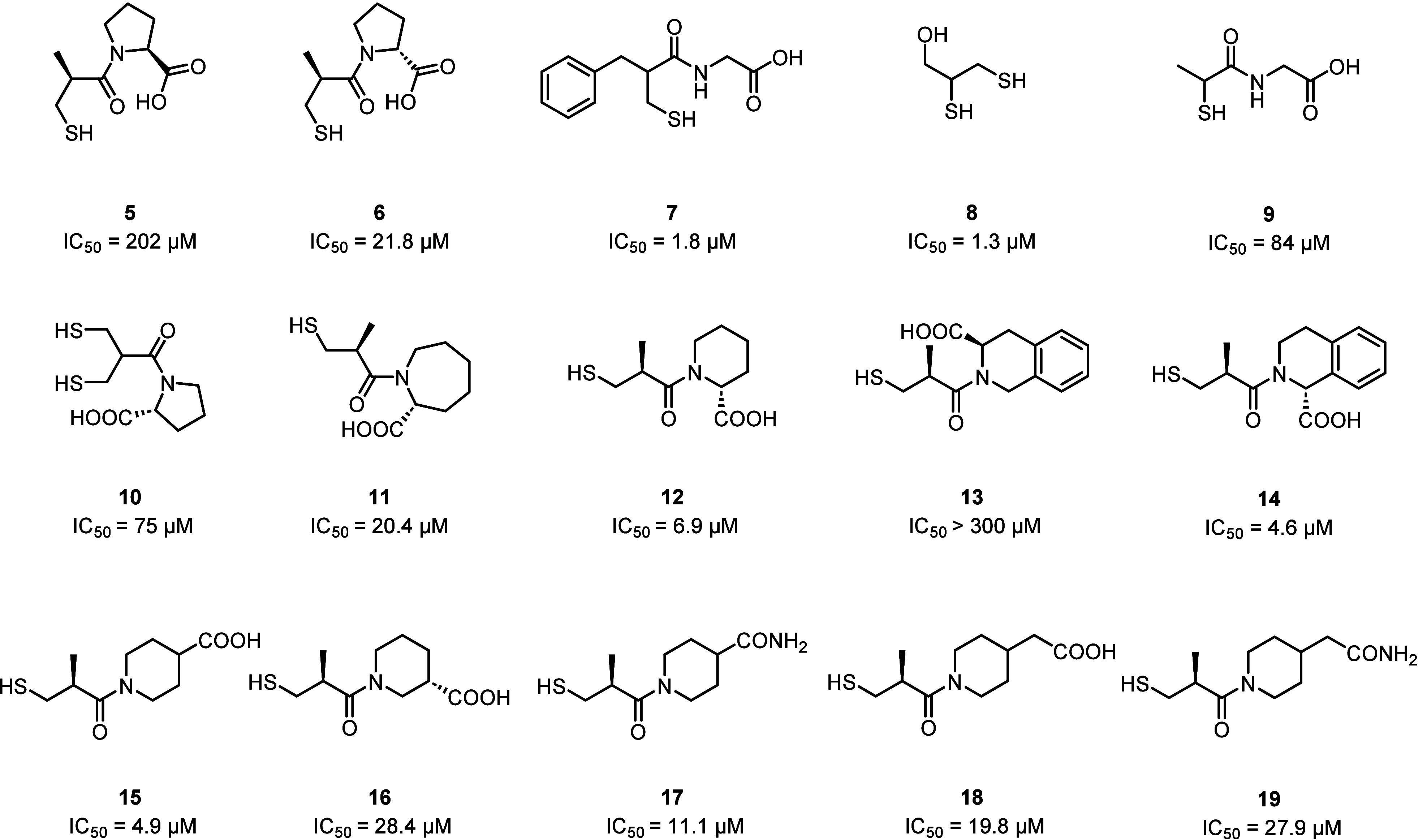
Structures of the representative thiol-containing compounds proposed as MBLi. IC_50_ are reported for NDM-1.

The ACE inhibitory activity is attributed to the ability of the thiol group to chelate the zinc ion in its active site. Later on, this molecule was also shown to be capable of inhibiting MBL subclasses B1–B3 as well, based on a structural comparison between the active sites of MBLs and ACE (Figure 12).^55^

**Figure 12:**
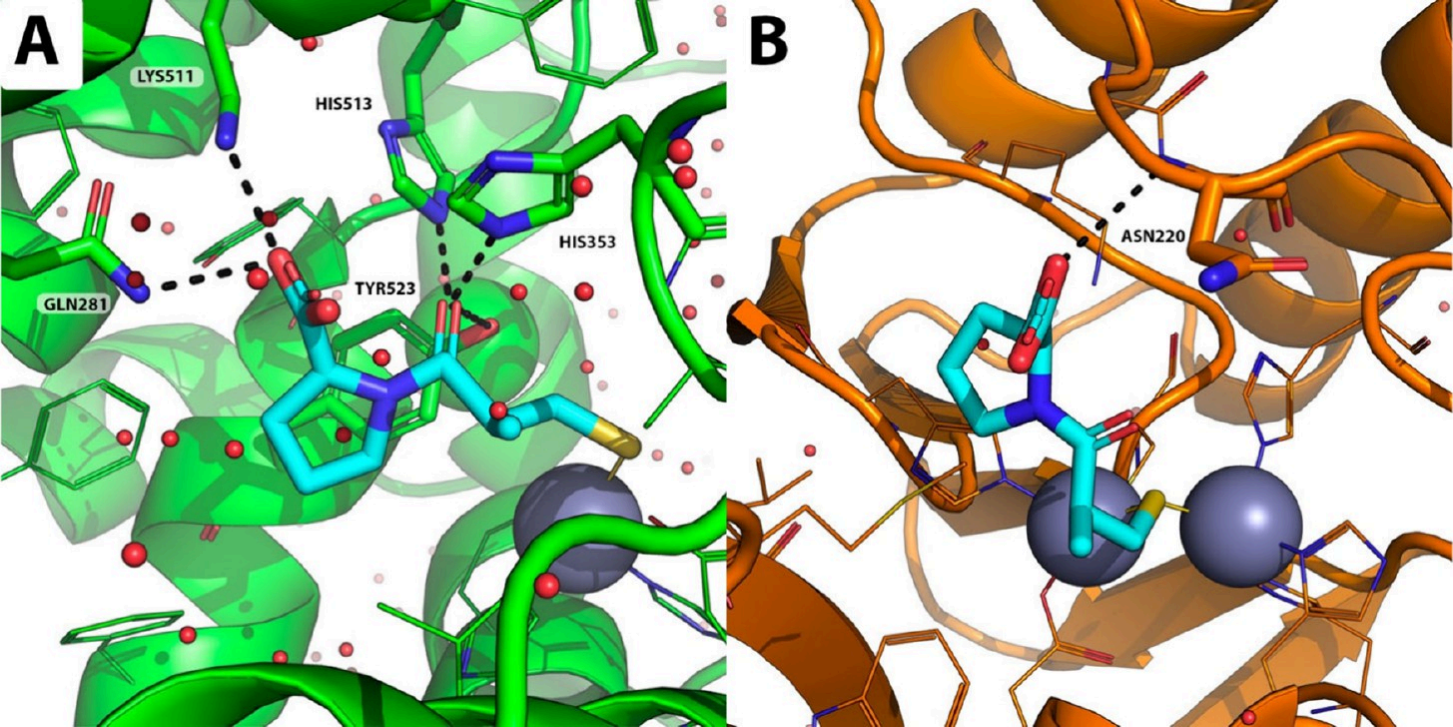
Comparison of the crystallographic binding mode of L-captopril in ACE and NDM-1 in complex with L-captopril. A) X-ray crystallography structure of human testicular angiotensin I-converting enzyme (ACE) (green cartoon, lines and sticks) in complex with L-captopril (cyan sticks) PDB-ID: 1UZF.^56^ B) X-ray crystallographic structure of *K. pneumoniae* NMD-1 (orange cartoon, lines and sticks) in complex with L-captopril (cyan sticks), PDB-ID: 4EXS.^57^ Polar interactions are highlighted by black dashed lines. Zinc ions are represented as gray spheres, while crystallographic water molecules are shown as small red spheres. Residues within 6 Å from the cocrystallized ligand are shown as lines; those involved in binding to L-captopril are shown as sticks.

The first crystal structure of L-captopril in complex with NDM-1 was solved by King et al. The L-captopril S1 atom has shown to insert between Zn1 and Zn2 (2.1 Å from both ions), displacing the nucleophilic water molecule and leading to a competitively inhibited enzyme; the hydrophobic face interacts with the L3 and L5 loops (V73 and M67 on the L3 loop interact with the L-captopril C6 and C3 atoms, while W93 on the L5 loop interacts with L-captopril C3 and C5 atoms.); the hydrophilic face interacts through H-bonds with N220 on the L10 loop, which interacts also with the 2 oxygens of the L-captopril carboxylate through a H-bond.^57^ Moreover, the (2*S*, 2*R*)-stereoisomer of L-captopril, containing a d-proline instead of an l-proline moiety and commonly known as D-captopril (**6**; Figure 11), has proven to be more active against some MBLs (e.g., NDM-1) than the commercial drug (IC_50_ = 21.8 μM and *K*_*i*_ = 1.3 μM for D-captopril versus IC_50_ = 202.0 μM and *K*_*i*_ = 3.9 μM for L-captopril).^57,58^ The binding modes of L-captopril and D-captopril into the active site of NDM-1 are almost identical, displaying a superimposable pattern in terms of hydrophobic and H-bond interactions (Figure 13).

**Figure 13:**
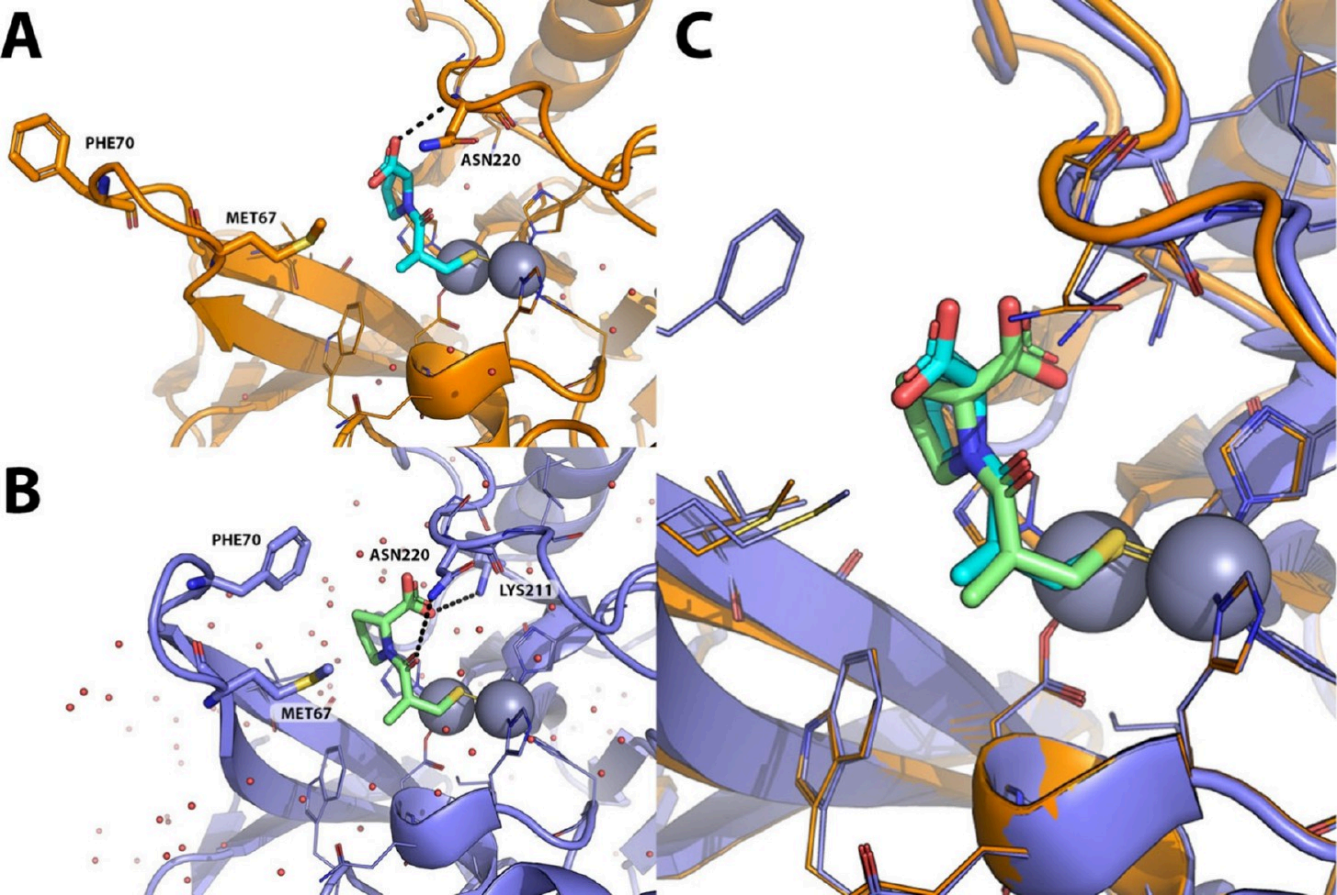
X-ray crystallography comparison of the binding mode of L- and D-captopril to NDM-1. A) X-ray crystallography structure of *K. pneumoniae* NDM-1 (orange cartoon, lines and sticks) in complex with L-captopril (cyan sticks) PDB-ID: 4EXS.^57^ B) X-ray crystallographic structure of NMD-1 (purple cartoon, lines and sticks) in complex with D-captopril (green sticks), PDB-ID: 5ZJ2.^58^ C) Superposition of NDM-1 structures in complex with L- and D-captopril. Polar interactions are highlighted by black dashed lines. Zinc ions are represented as gray spheres, while crystallographic water molecules are shown as small red spheres. Residues within 6 Å from the cocrystallized ligands are shown as lines; those involved in binding to L- and D-captopril are displayed as sticks.

A different orientation of the proline ring moiety allows further hydrophobic interactions with M67 and especially F70 residues in the L3 loop, thus inducing a more closed receptor conformation. Moreover, the opposite orientation of the carboxylate group allows one more H-bond interaction with a water molecule that stabilizes K211 in the L10 loop, where the K211 residue plays an important role in β-lactam hydrolysis by NDM-1, orienting the negatively charged carboxylate group of β-lactam substrates during their binding to the active site. Both these two additional interactions increase the binding of D-Captopril with the active site of NDM-1 and contribute to its higher potency.^58^ However, the blood-pressure-lowering effect of captopril is not desirable when treating a bacterial infection. Again, driven by a repositioning activity, other thiol-containing drugs, potentially showing comparable binding modes, were assessed. Among them, thiorphan (**7**), dimercaprol (**8**), and tiopronin (**9**) (Figure 11) showed the most promising MBL inhibitory potential,^51^ with subsequent cocrystallization in NDM-1 confirming a binding mode superimposable to captopril (Figure 14).^59^

**Figure 14:**
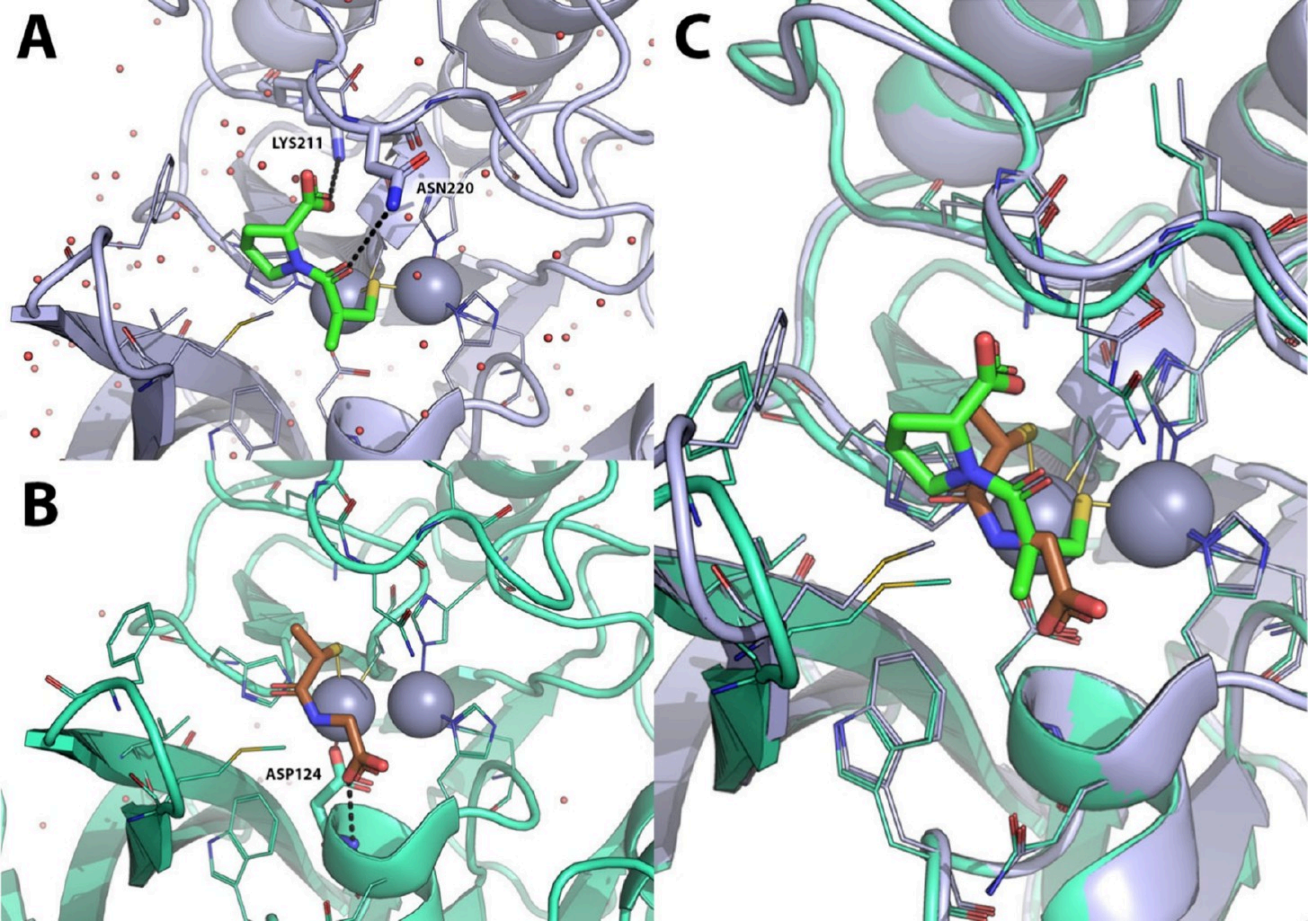
X-ray crystallography structure of *K. pneumoniae* NDM-1 in complex with D-captopril and Tiopronin. A) X-ray crystallographic structure of NMD-1 (violet cartoon, lines and sticks) in complex with D-captopril (green sticks), PDB-ID: 5ZJ2.^58^ B) X-ray crystallographic structure of NMD-1 (green-cyan cartoon, lines and sticks) in complex with Tiopronin (brown sticks), PDB-ID: 5A5Z.^60^ C) Superimposition of the two X-ray crystallography structures with magnification of the ligand binding poses. Polar interactions are highlighted by black dashed lines. Zinc ions are represented as gray spheres, while crystallographic water molecules are shown as small red spheres. Residues within 6 Å from the cocrystallized molecules are shown as lines; those involved in binding to the ligands are shown as sticks.

Unfortunately, to date, none of these compounds have reached clinical trials, despite the good inhibition profile in enzymatic assays. One of the main reasons, as proposed by Rotten et al., is the high lipophilicity displayed by these compounds, which limits the transport of the inhibitor through the outer membrane of Gram-negative pathogens and the accumulation of the compounds in the periplasmic space, in which the MBLs are located.^53,61^

Subsequently, starting from the complex structure of NDM-1/D-captopril, Ma et al. developed novel compounds through modifications at different sites of D-captopril. These modifications were applied on (i) the methyl group (**10**), (ii) the ring size (**11** and **12**), (iii) the hydrophobicity of the ring structure (**13** and **14**), and (iv) the position of the carboxylate group (**15–19**) (Figure 11). Most of the compounds thus obtained interact with the active site of NDM-1 intercalating the thiol group between the two zinc ions (such as captopril) and through hydrophobic interactions between the ring structure and the L3 loop. Moreover, the 6-membered ring structure bearing a carboxylate group at the 2- or 4- position (**12** and **15**, respectively) is responsible for a high inhibition potency toward NDM-1, although the highest *in vitro* inhibition potency is obtained when the 6-membered ring is connected to an additional phenyl ring (**14**). The potential synergistic activity of the most promising captopril derivatives (namely, **12**, **14**, and **15**) was tested in combination with Meropenem against NDM-1 producing *E. coli* strains. While compounds **12** and **15** showed a significant reduction of Meropenem MIC, compound **14** showed a lower inhibitory effect at the cellular level, probably attributable to the lack of suitable properties necessary to readily diffuse through the bacterial outer membrane and reach the periplasmic space.^58^

#### 5.2.2 Carboxylic Acids

Carboxylic acid derivatives, containing a moiety derived from dipicolinic acid (DPA) in their structure, act as MBLi through coordination between the carboxylic acid group and the zinc ions in the active site of these enzymes. Moreover, the spectroscopic analysis also revealed that the DPA analogues can form a stable ternary complex with NDM-1.^51^ Among the designed compounds, the biaryl-DPA ones were very powerful (compound **21**; Figure 15), since the addition of hydrophobic substituents on the DPA core can enhance the interactions with the hydrophobic surface on the β-hairpin loop close to the active site of NDM-1 (Figure 16).

**Figure 15:**
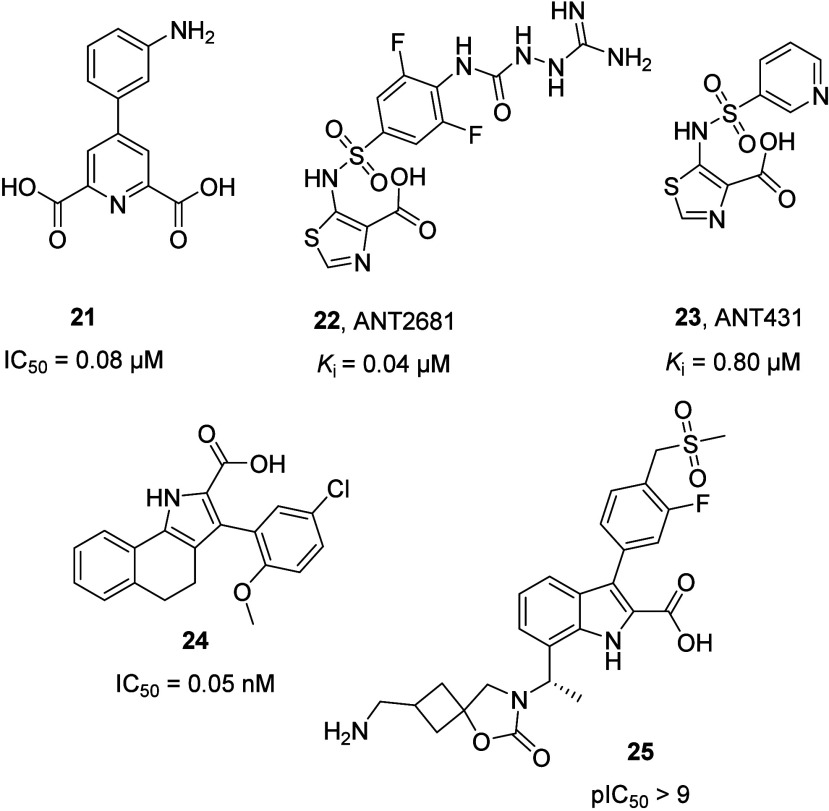
Chemical structure of NDM-1 inhibitors containing a carboxylic group. IC_50_, *K*i, and pIC_50_ values are reported for NDM-1.

**Figure 16:**
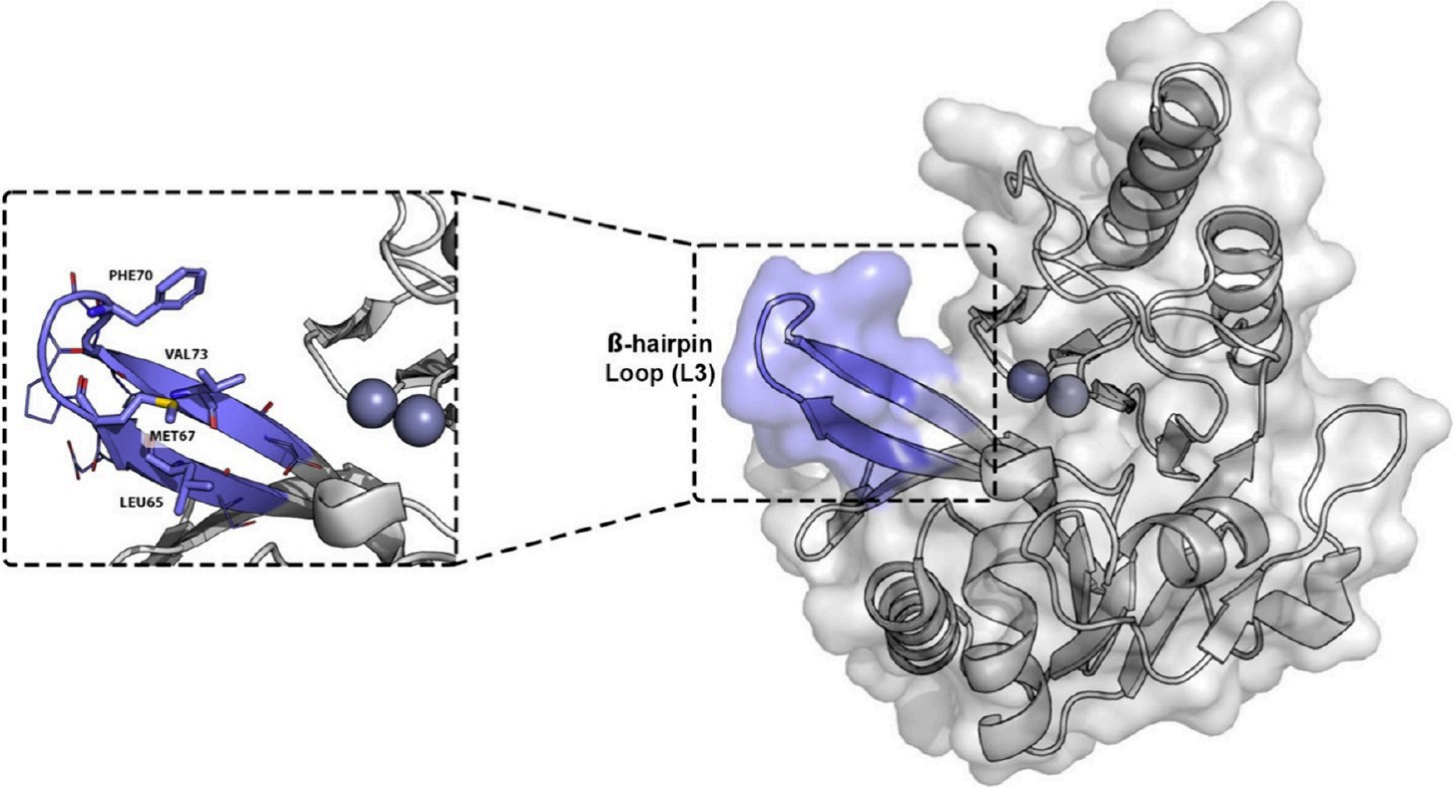
β-Hairpin loop (L3 loop) in the X-ray structure of *K. pneumoniae* NDM-1 (PDB-ID: 3SPU, chain A).^46^ The L3 loop is represented as a purple cartoon and transparent surface; the remaining part of the protein is shown as a transparent surface and cartoon. Zinc ions are represented as gray spheres. Amino acids of the L3 loop are represented as lines, while those that compose the hydrophobic region of the L3 loop are represented as sticks.

In *in vivo* assays, these derivatives have demonstrated inhibitory capacity on NDM-1 by restoring the activity of imipenem in strains of NDM-1 producing *E. coli.* ^51^ Recently, compound ANT2681 (**22**; Figure 15), a novel thiazole-carboxylate inhibitor optimized from ANT431 (**23**; Figure 15), reached the preclinical phase, due to its promising results both in *in vitro* and *in vivo* assays.^62,63^ Further discussion on compounds **22** and **23** is given below. Most recently, starting from picolinic acid and by applying scaffold hopping, conformation constrained, and substituent-decorating strategies, Dihman et al. synthesized novel dihydrobenzo indole (dBI) derivatives as a new class of potent MBLi. Among these, compound **24** (Figure 15) exhibits the best inhibitory activity against MBLs, with acceptable physicochemical and ADME properties. Given their potent inhibitory activity, these compounds emerge as compelling leads for future preclinical and clinical investigations.^64^ Indole-2-carboxylates (InC) represent another class of inhibitors showing promising inhibitory activity against all major clinically relevant MBLs. This class, based on indole-2-carboxylates (InCs), was identified by Schofield, Brem, and co-workers in 2021, through a high-throughput screening (HTS) of a compound library, followed by the optimization of the hits.^48^ These compounds are characterized by a broad-spectrum of action against MBLs and a good safety profile on mouse infection models. Among these, compound **25** (Figure 15) shows a high potency inhibition against all three types of B1MBLs, with pIC_50_ values >9 for NDMs and VIM and >7 for IMP-1.^65^

The SAR studies carried out on the InCs have revealed the importance of the InC indole NH, C2 carboxylate, and C3 and C7 alkyl and/or aryl groups for the potent MBL inhibition:^65^ these functional groups are responsible for a binding mode of these compounds to the β-lactamases that resembles the binding mode of both the intact substrate (a) and substrate-derived product (b) to B1MBLs:

(a) the binding of C7, C3 alkyl or aryl, and C2 carboxylate substituted InCs mimics those of β-lactams;^65^
(b) The indole NH forms a hydrogen bond with the bridging water/hydroxide, which resembles the protonation of the β-lactam nitrogen during hydrolysis (necessary for the hydrolytic mechanism). Plus, the InCs-enzyme complex resembles the carbapenem-derived products bound to the MBL active site, in particular in their enamine tautomeric form, which represents the main product of MBL-catalyzed carbapenem hydrolysis.^65,66^

This binding mode led us to hypothesize an unprecedented mechanism of action: these compounds seem to lock the zinc-complexed hydroxide rather than displacing it. This is further supported by the identical distances between the two zinc ions in both the intact enzyme and the enzyme with the inhibitor bound (Zn–Zn distance of 3.5 Å).^48^ This mechanism is mainly due to the indole NH-bridging water/hydroxide interaction and the C7 alkyl group enclosing the bridging water/hydroxide. Moreover, they are weak metal ion chelators, which means they lack all the side effects associated with strong chelators.^65^ The *in vivo* efficacy of InC **25** was assessed in multiple murine peritonitis/sepsis and thigh models, using four different carbapenem-resistant extensively drug-resistant strains (three *E. coli* and one *K. pneumoniae*). In these murine infection models, a single dose of **25** (10 mg kg^–1^) plus Meropenem (16–90 mg kg^–1^) reduced the bacterial load up to a 7-log fold. Regarding *in vivo* safety, InC **25** showed no interaction with >65 human receptors and a good tolerance profile. Macroscopic organ changes were ruled out, and low levels of plasma and urine markers of kidney and liver damage were observed, thus highlighting a favorable toxicity profile for the compound. These results lead to consider compounds belonging to InCs as suitable starting points for clinical development of synergistic agents to be combined with β-lactam antibiotics.^65^

#### 5.2.3 Cyclic Boronates

The boronate class of MBLi can be clustered into mono- and bicyclic boronates. It is important to highlight that cyclic boronates were originally conceived as effective inhibitors of the SBL class of beta lactamases.^67^ Among the monocyclic boronates, the most important is vaborbactam (**26**; Figure 17), an SBLi that is the first representative of this class receiving FDA approval. Although lacking MBLs inhibitory activity, vaborbactam was used as a starting point to develop novel broad-spectrum SBLi and MBLi. Accordingly, follow up studies disclosed three novel bicyclic boronates, namely taniborbactam, xeruborbactam, and KSP-1007 (**27, 28,** and **29**, respectively; Figure 17) which showed a dual inhibitory profile toward several SBL and MBL isoforms.^51,68^

**Figure 17:**
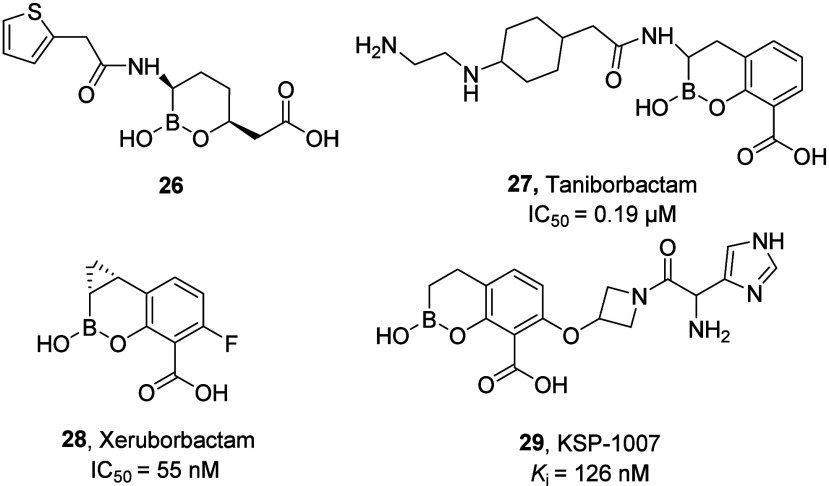
Chemical structure of NDM-1 inhibitors with a cyclic boronic structure.

Most specifically, these bicyclic boronates mimic the high-energy-state tetrahedral transition state, displacing the Zn­(II)-bound water molecule (Figure 18).^48^

**Figure 18:**
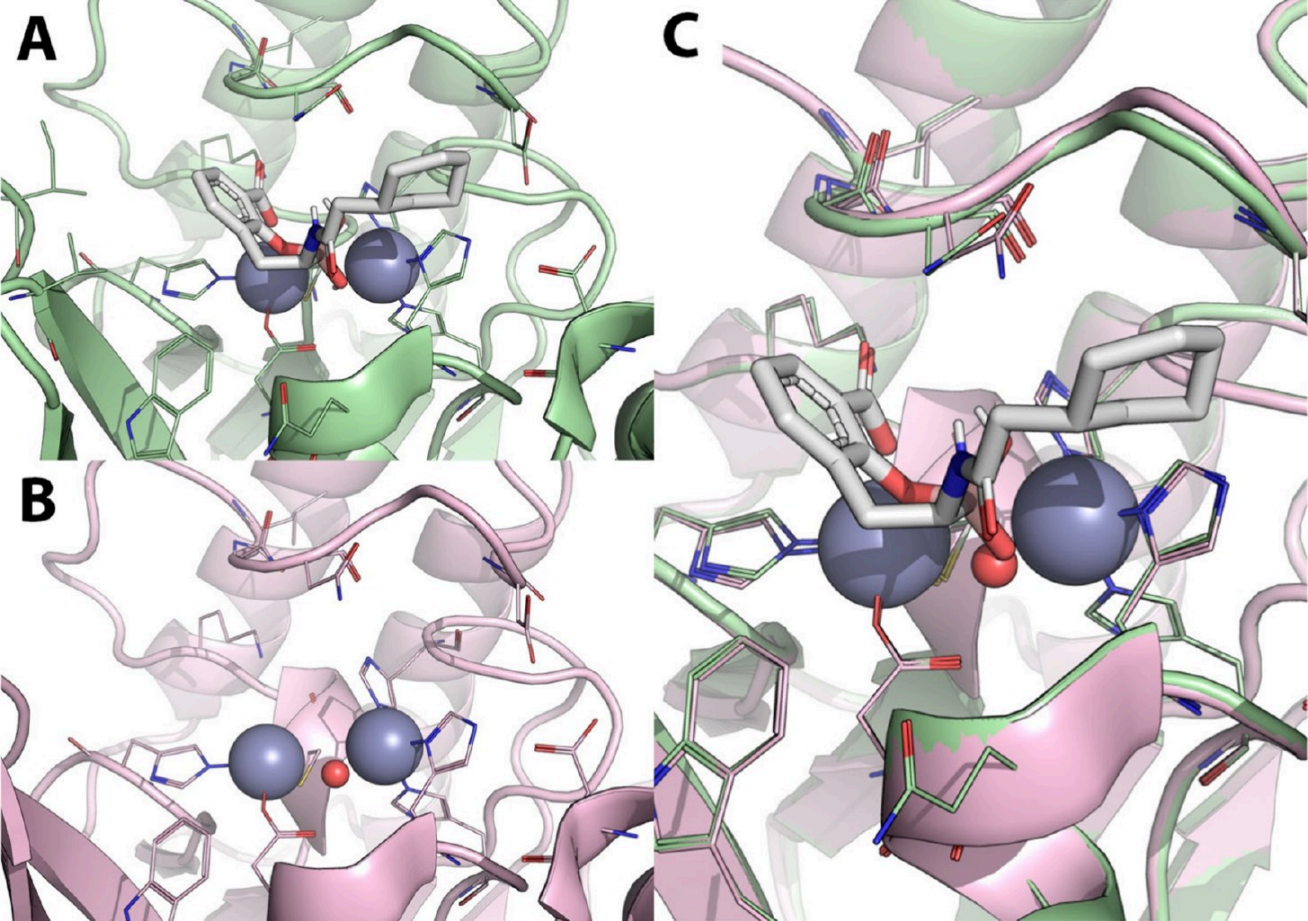
Displacement of the Zn­(II)-bound water molecule by taniborbactam. A) X-ray crystallographic structure of *K. pneumoniae* NMD-1 (green cartoon and lines) in complex with taniborbactam (gray sticks), PDB-ID: 6RMF.^69^ B) X-ray crystallographic structure of *K. pneumoniae* NMD-1 (pink cartoon and lines) in complex with (2*R*,4*S*)-5,5-dimethyl-2-[(1*R*)-1-(2-naphthalen-1-yloxyethanilamino)-2-oxidiethyl]-1,3-thiazolidine-4-carboxylic acid (PDB-ID: 8I8F).^70^ For the sake of clarity, the cocrystallized ligand has been removed from PDB-ID: 8I8F in panel B. Zinc ions are represented as gray spheres, and residues within 6 Å from taniborbactam are shown as lines.

While xeruborbactam is currently in phase I, taniborbactam and KSP-1007 have already completed phase III and phase I clinical trials, respectively. Further discussion on compounds **27**, **28**, and **29** will be provided below. Inspired by structural features and the mechanism of action of taniborbactam, Gulyás et al. have recently designed dynamically chiral phosphonic acids as novel potential MBLi. As a unique feature, these compounds exhibit the remarkable ability to bind MBLs with both of their stereoisomeric forms. This structural adaptability enables broader inhibitory activity across diverse enzyme variants and may significantly reduce bacteria’s ability for resistance development.^71^

#### 5.2.4 Biphenyl Tetrazole Derivatives

In 2022, Mandal and his co-workers from Merck identified compound **30** (Figure 19) as a novel pan-MBLi candidate. This compound was developed through optimization of compound **31** (Figure 14), a newly discovered NDM-1 inhibitor. Further optimizations led to compound **32** (Figure 19), an early broad spectrum MBLi.

**Figure 19:**
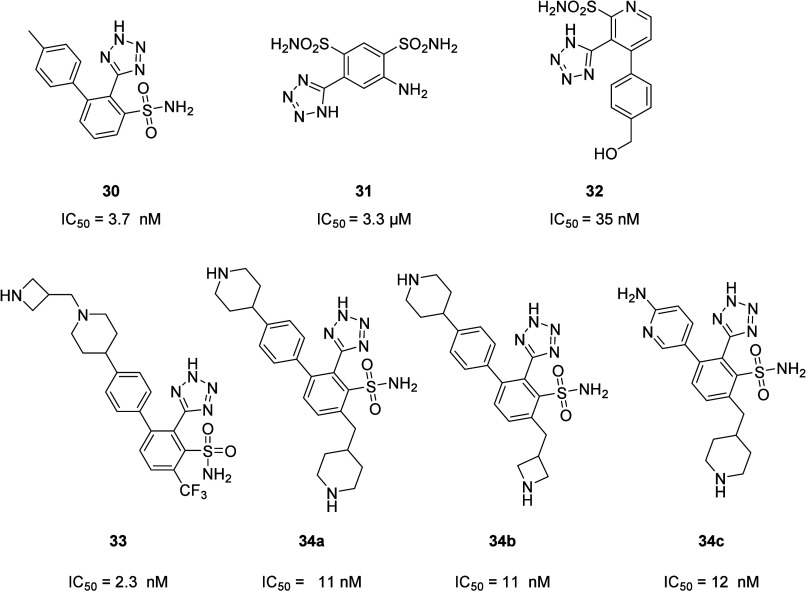
Chemical structures of the early broad spectrum-MBLi **30**, its precursor **31**, and its derivatives **32**, **33**, and **34a**–**c**. IC_50_ are reported for NDM-1.

Compound **30** inhibits MBLs by chelating the zinc ions through its tetrazole and sulfonamide moieties (in particular, the first coordinates with Zn2, while the second coordinates with Zn1 and Zn2), demonstrating specific binding to metal ions in MBLs (Figure 20).

**Figure 20:**
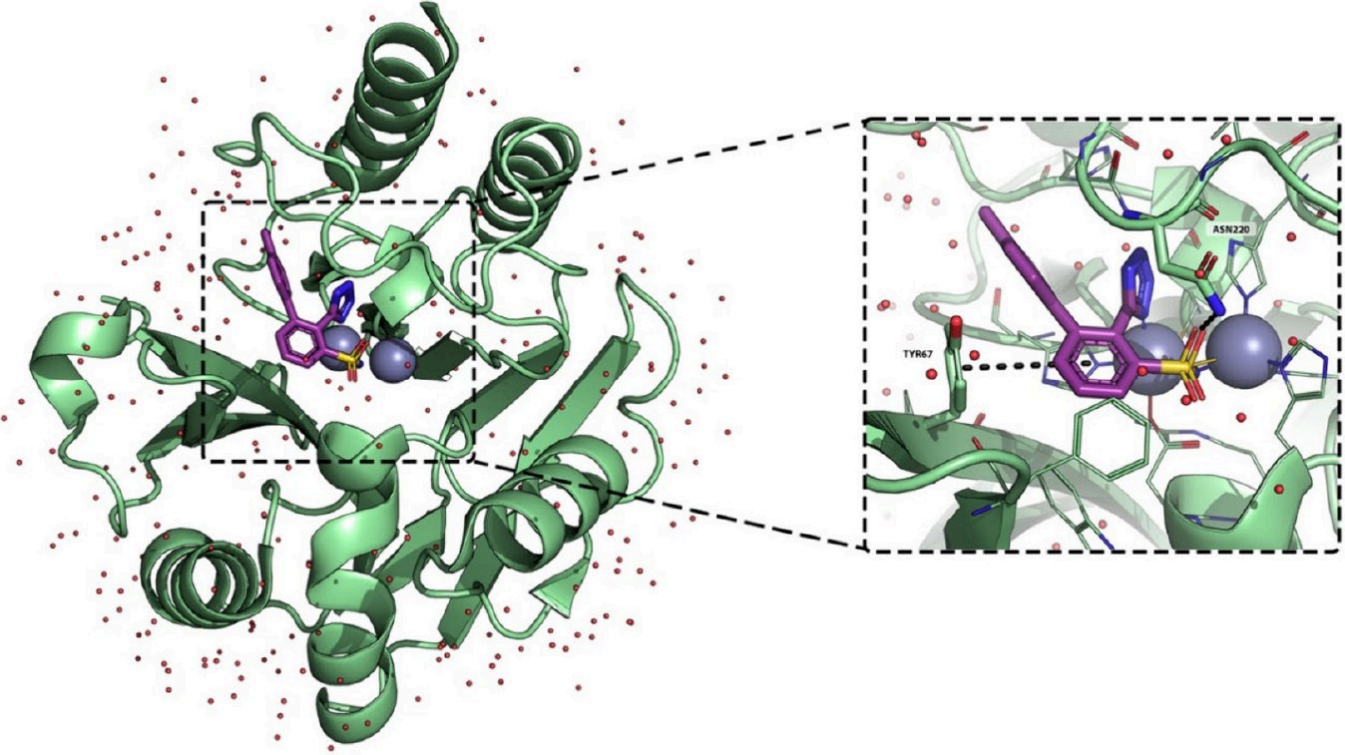
X-ray crystallography structure of *P. aeruginosa* NDM-1 in complex with molecule **30** (PDB-ID: 7UP2).^72^ NMD-1 is shown as a green cartoon and lines; molecule **30** is shown as purple sticks. Zinc ions are represented as gray spheres, while crystallographic water molecules are shown as small red spheres. Polar interactions are highlighted by black dashed lines, residues within 6 Å from the cocrystallized ligand are shown as lines, and those involved in binding to **30** are shown as sticks.

Later on, compound **30** was combined with a hydroxyl methyl and a pyridine, resulting in a new subclass of compounds (pyridine derivatives), to which compound **32** belongs. These modifications led, on one side, to a loss in potency against MBLs but, on the other side, to an improvement in the MITC_95_ (minimum concentration of MBLi required to inhibit bacterial growth by 95%) (Table 3). This improvement has been attributed to the enhanced accumulation of these compounds in the periplasmic space, where MBLs are located.^72^ Indeed, introducing polar and basic functional groups improve the Gram-negative bacterial cell penetration.^73^ In particular, starting from compound **30**, compounds **33** and **34a**–**c** (Figure 19) were obtained. The *in vivo* evaluation of these compounds confirmed that adding basic amines is essential to counteract *P. aeruginosa* efflux issues and increase Gram-negative cell accumulation. But, on the other hand, the increase of the cationic nature of the molecules is also responsible for mast cell degranulation, with histamine release and severe anaphylactoid reactions in rats and dogs. Therefore, this evidence represents the starting point for the development of new broad-spectrum MBLi with improved Gram-negative bacterial cell penetration.^74^ In *in vivo* studies, compound **31**, in combination with imipenem, has been demonstrated to strongly reduce the bacterial burden, in both the spleen and kidney, in a murine infection model.^72^

**Table 3:** Comparison of IC_50_ (nM) and MITC_95_ (μM) of Compounds **30** and **32**

	Enzyme IC_50_ (nM)			MITC_95_ (μM)^b^		
Cpd^a^	NDM-1	IMP-1	VIM-1	EC^c^	SM^d^	KP^e^
**30**	3.7	64	57	1.48	13.8	127
**32**	35	369	269	0.558	5.19	11.76

a No intrinsic antibacterial activity was seen in the absence of IPM.

b Minimum concentration of MBLi required to inhibit 95% growth in the presence of 4 μg/mL IPM.

c Minimum concentration of IPM alone required to inhibit 95% growth is 32 μg/mL.

d Minimum concentration of IPM alone required to inhibit 95% growth is 16 μg/mL.

e Minimum concentration of IPM alone required to inhibit 95% growth is 64 μg/mL; EC, *Escherichia coli* expressing NDM-1; SM, *Serratia marcescens* expressing IMP-1; KP, *Klebsiella pneumoniae* expressing VIM-1.^72^

### 5.3 Metallodrugs

Colloidal bismuth subcitrate (CBS) (**35**; Figure 21) and related Bi­(III) compounds irreversibly inhibit different types of MBLs by replacing with Bi­(III) one of the two zinc ions, as revealed by X-ray crystallography.^48^ CBS has been demonstrated to restore Meropenem efficacy against MBL-positive bacteria *in vitro* and in a mice infection model. IC_50_ values were determined for NDM-1, VIM-2, and IMP-4 (2.81, 3.54, and 0.70 μM, respectively). Moreover, it also slows the development of higher-level resistance in NDM-1-positive bacteria. Therefore, these Bi­(III) compounds have demonstrated a high potential to become the first broad-spectrum B1MBLi to treat MBL-positive bacterial infection in conjunction with existing carbapenems (see Table 4).^75^

**Figure 21:**
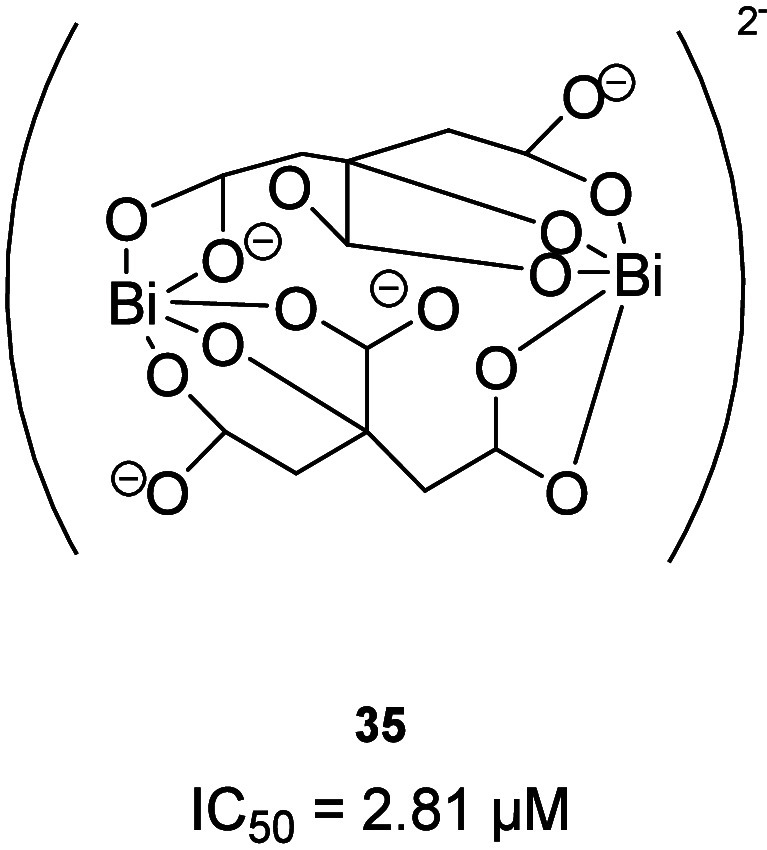
Chemical structure of CBS.

**Table 4:** Summary Table of MBL Classes

MBLi class	Representative compounds	Mechanism Of Action
**Strong Chelating Agents**	AMA, EDTA,NOTA, DOTA	Coordination and removal of Zn ions from the active site
**Thiol-containing Compounds**	captopril, thiorphan, dimercaprol, tiopronin	Displacement of Zn-complexed hydroxide/water molecule
**Carboxylic Acids**	ANT2681, ANT431	Displacement or locking of Zn-complexed hydroxide/water molecule
**Cyclic Boronates**	taniborbactam, xeruborbactam KSP-1007	Displacement of Zn-complexed hydroxide/water molecule
**Biphenyl Tetrazole Derivatives**	-	Displacement of Zn-complexed hydroxide/water molecule
**Metallodrugs**	CBS	Replacement of Zn ions and irreversible inhibition

### 5.4 MBLi: Preclinical and Clinical Progress

To date, only four compounds displaying MBL inhibitory activity are undergoing preclinical or clinical trials (Table 5), namely, compounds ANT2681 (**22**), taniborbactam (**27**), xeruborbactam (**28**), and KSP-1007 (**29**).

**Table 5:**
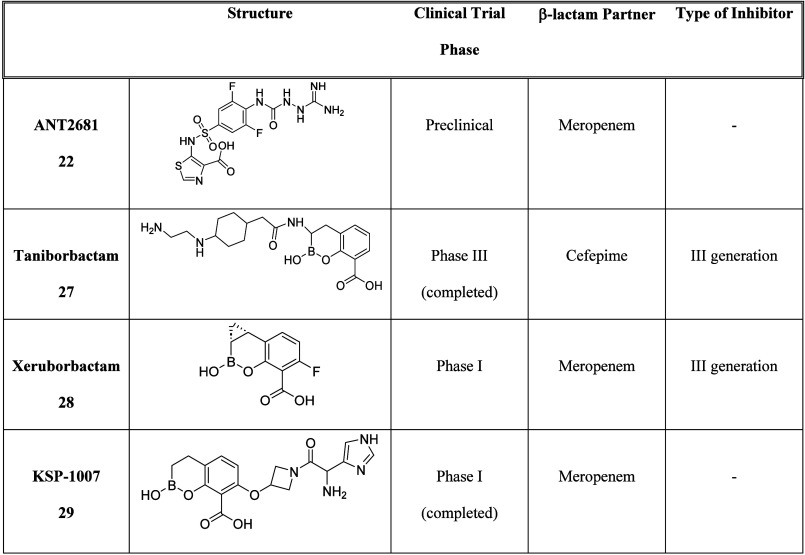
Summary Table of β-Lactam-MBLi Combinations Currently in Preclinical and Clinical Trials

Compound ANT2681 (**22**) is a novel thiazole-carboxylate inhibitor currently undergoing preclinical development to combine it with meropenem as a new treatment for serious infections caused by MBL-producing CRE. It displays the highest affinity for NDM-1, lower affinity for VIM-1, and very poor affinity for IMP-1 and has shown efficacy in a mouse thigh model with an NDM-1-producing clinical isolate of *K. pneumoniae*. ANT2681 has been optimized from ANT431 (**23**) – which demonstrated efficacy in a mouse infection model – and is the result of a medicinal chemistry hit-to-lead program starting from pyridine-2-carboxylic acid (picolinic acid).^62,63^ ANT2681 inhibits MBLs through interaction with the zinc ions in the active site of these enzymes and mimicking the enzyme–product complex,^65^ which leads to a competitive noncovalent inhibition.^76^ Taniborbactam (VNRX-5133) (**27**), characterized by a broad spectrum of inhibitory activity against KPC, OXA-48, and MBLs (such as VIM and NDM but not IMP),^77^ is the first boronate inhibitor able to inactivate both SBLs and MBLs enzymes through different mechanisms.^78^ While vaborbactam, a SBLi, can only inhibit SBLs, its addition with an aromatic group and a carboxylic acid on the boronate ring leads to taniborbactam, able to bind MBL enzymes too.^31^ Taniborbactam completed phase III clinical trials (cefepime – taniborbactam combination) to investigate its safety and efficacy against complex urinary tract infection (UTI), resulting superior to meropenem in patients with complicated UTIs.^62,79^ Despite promising results, in 2024 the US FDA rejected the New Drug Application (NDA) for the cefepime-taniborbactam combination due to manufacturing issues, also requesting additional data on the drug’s chemistry, manufacturing, and controls.^80^ This rejection highlights the significant challenges continuing to hinder the ongoing MBLi development and further slow their introduction into clinical practice. Xeruborbactam (QPX7728) (**28**) is characterized by a ultrabroad-spectrum activity against all classes of β-lactamases.^81^ In comparison to taniborbactam, the introduction of a cyclopropyl group increases the hydrophobic interaction in the active site and, as a consequence, the inhibitory activity. Currently, xeruborbactam is in phase I clinical trials (meropenem – xeruborbactam combination) to evaluate its safety, the pharmacokinetics of intravenous treatments, and to develop the orally administered forms.^31,62^ KSP-1007 (**29**), a novel bicyclic boronate-based β-lactamase inhibitor, has completed phase I clinical trials (meropenem – KSP-1007 combination) to treat infections caused by carbapenem-resistant Gram-negative bacteria. It effectively inhibits all classes of β-lactamases and has shown potent activity in murine models of systemic, urinary tract, and thigh infections.^68^

## 6 Current Strategies for Enhancing *in Vivo* Activity of MBLi

MBLi proposed so far demonstrated good enzymatic activity; yet their effectiveness at the cellular level is still compromised by an insufficient cell penetration across the outer membrane of Gram-negative bacteria, coupled with the action of active efflux pumps, both preventing them from accumulating in the periplasmic space.^74^ Current strategies to potentially overcome these challenges and enhance periplasmic accumulation of MBLi can be summarized as follows: (i) modification of the physicochemical properties of MBLi through the addition of specific functionalities,^73^ (ii) the development of molecular Trojan horses,^82^ and (iii) the development of nanoparticle-based delivery systems for MBLi.^83^

### 6.1 Optimizing Cellular Accumulation by Refining Physicochemical Properties of MBLi

In 2008, O’Shea and Moser highlighted that high polarity and positive charges are critical for enhancing cellular accumulation,^84^ with basic amines, particularly primary ones, being the most effective structural motif.^74^ This is determined by the hydrophilic nature of the outer membrane lipopolysaccharides and of the porin channels,^85^ as well as by the structure of the efflux pumps,^86^ leading to increased uptake and reduced efflux of the MBLi. This strategy was the one exploited by Mandal and his co-workers while optimizing the *in vivo* properties of the novel developed biphenyl tetrazole derivatives, as mentioned in paragraph 5.2.4.^72^

### 6.2 The Trojan Horse Mechanism of Delivering Antibiotics

The **Trojan Horse strategy provides a new approach to drug delivery by improving** cellular uptake of antibiotics and enhancing target selectivity, conjugating the drugs with compounds of specific interest for the microbe, such as siderophores or other molecules resembling their natural substrates.^87^ Given its advantages, this strategy has been applied in the development of new MBLi. Liu et al. synthesized a set of molecular Trojan horses selectively targeting NDM-1 by incorporating the activated form of ebselen (**36**; Figure 22) (a covalent MBLi) into 7-aminocephalosporanic acid derivatives through a C–Se bond. Since the C–Se bond is more stable than the N–Se bond in ebselen, the cephalosporins (targeted carrier portion of these molecules) are hydrolyzed by NDM-1, thus releasing the active form of ebselen directly into the catalytic site of the enzyme and enhancing the target specificity of the molecules. Structural modifications were then applied to the cephalosporin portion in order to increase the compounds’ affinity for NDM-1. Later, the most promising compounds were selected for further investigation in *in vitro* and *in vivo* assays, evaluating their synergistic antibacterial activity with MEM. Among these, compound **37** (Figure 22) was identified as the optimal candidate, showing an IC_50_ value of 7.03 μM, an excellent synergistic antimicrobial activity (MIC of MEM reduced by 4- to 32-fold), an exceptional *in vitro* safety and metabolic stability, and, most importantly, inhibition of growth and reproduction of *E. coli* ZJ487 strain in mice when combined with MEM.^82^ Another widely explored Trojan horse approach is the linkage of antibiotics to siderophores – high-affinity iron chelators – forming complexes known as sideromycins that exploit the iron-siderophore uptake system, thus counteracting the low outer-membrane permeability of bacteria and enhancing cellular accumulation.^87^ Siderophore-conjugated monocyclic β-lactams, such as SC23 (**38**; Figure 22), U-78608, and BAL30072, have been widely studied over the past decade to develop synthetic sideromycins that combine the inherent hydrolytic stability of monocyclic β-lactams with the improved antibiotic uptake conferred by the siderophore moiety. SC23 was active against carabepemase and cephalosporinase producing *A. baumannii*. Unfortunately, the tedious synthetic processes and other limitations have prevented them from reaching clinical application.^88^ Despite this, the development of new siderophore-containing molecules for treating infections caused by resistant bacteria has advanced, inspired by the promising results of Cefiderocol (Fetroja) (**39**; Figure 22), which is a synthetic sideromycin developed by Shionogi & Company Ltd. and recently approved by the US FDA.^89^ Cefiderocol consists of a cephalosporin linked to a catechol moiety, which is responsible for iron chelation, making this drug highly effective against troublesome MDR bacteria (including carbapenemase-producing Enterobacterales).^61^ Starting from the aztreonam structure, Krajnc and Gobec synthesized three new compounds (**40–42**; Figure 22), respectively, in 4, 5, and 7 steps. These compounds showed a significant improvement in antimicrobial properties *in vitro* with high potential for further optimization.^88^ Moreover, Rotter et al. focused on the development of novel siderophore-containing MBLi, incorporating the catechol moiety into thiol-based MBL inhibitors.

**Figure 22:**
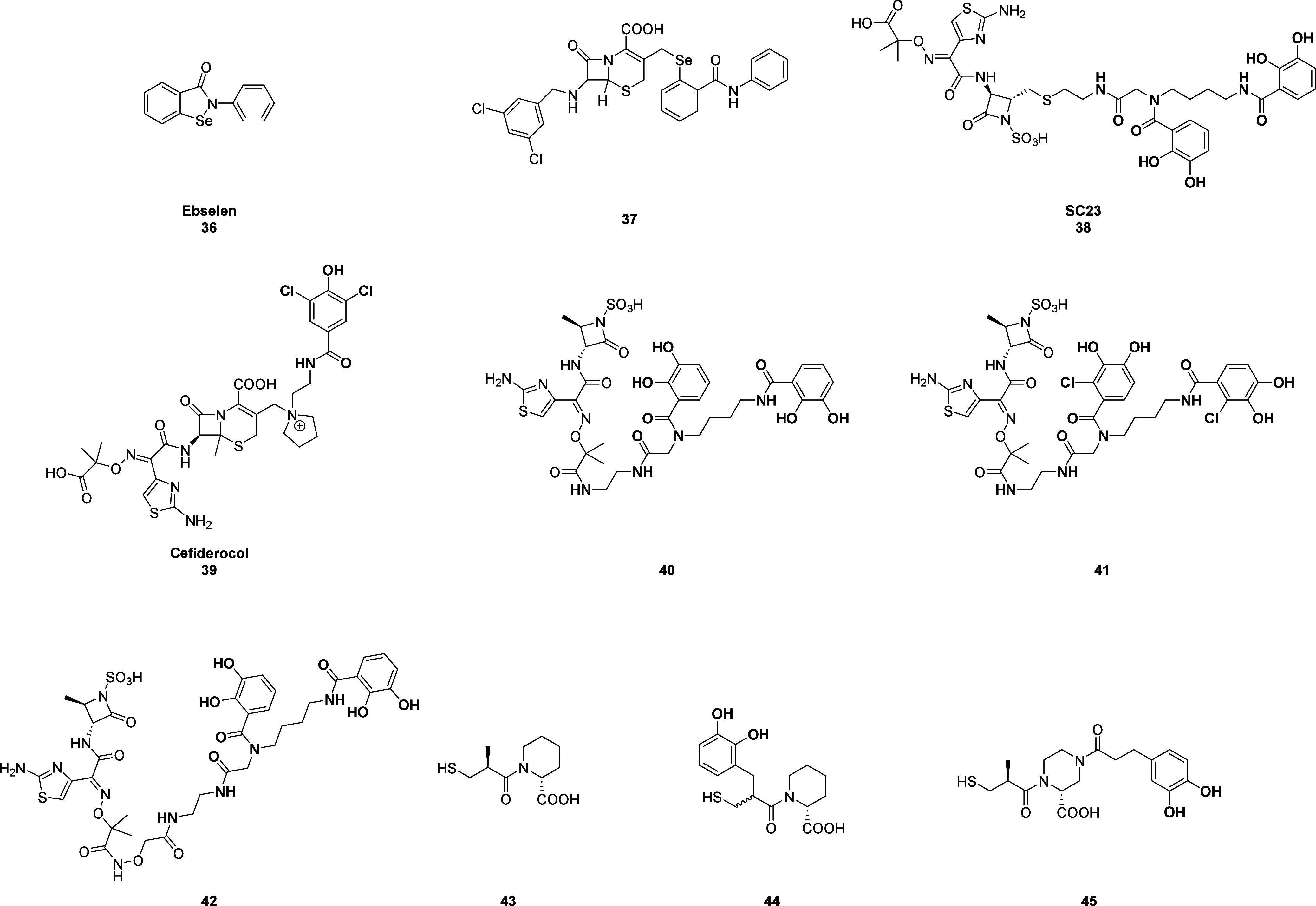
Structures of (i) the molecular Trojan horse (**37**) developed from ebselen (**36**), (ii) the siderophore-conjugated monocyclic β-lactams (**38** and **40–42**); (iii) cefiderocol (**39**), (iv) siderophore-Containing thiol-based MBLi (**44**, **45**) derived from (2*R*)-1-[(2*S*)-2-methyl-3-sulfanyl-propanoyl] piperidine-2-carboxylic acid (**43**).

They started from compound **43** ((2*R*)-1-[(2*S*)-2-methyl-3-sulfanyl-propanoyl] piperidine-2-carboxylic acid) (Figure 22), and considering its X-ray structure in complex with NDM-1 (Figure 23), they added a catechol moiety at the methyl position, thus obtaining compounds **44** and **45** (Figure 22), which were proposed as potential siderophore-containing MBLi. Compound **44** potently inhibited VIM-1 and IMP-7, but it failed to inhibit NDM-1 in the submicromolar range, while compound **45** inhibited all tested MBLs in the submicromolar range. Moreover, compound **45** exhibited a significant improvement in the MIC of imipenem in a clinical *K. pneumoniae* isolate, expressing NDM-1 at a concentration of 16 μg/mL. These results trigger the interest in developing novel siderophore-containing MBLi potentially characterized by an increased cellular accumulation.^61^ However, it is important to highlight that siderophore-antibiotic conjugates may also encounter resistance mechanisms, since the specific outer membrane transporters for siderophore-containing molecules (TonB-dependent receptors) may undergo specific mutations. Therefore, future perspectives will include the synthesis of siderophore-drug conjugates targeting multiple TonB-dependent receptors.^87^

**Figure 23:**
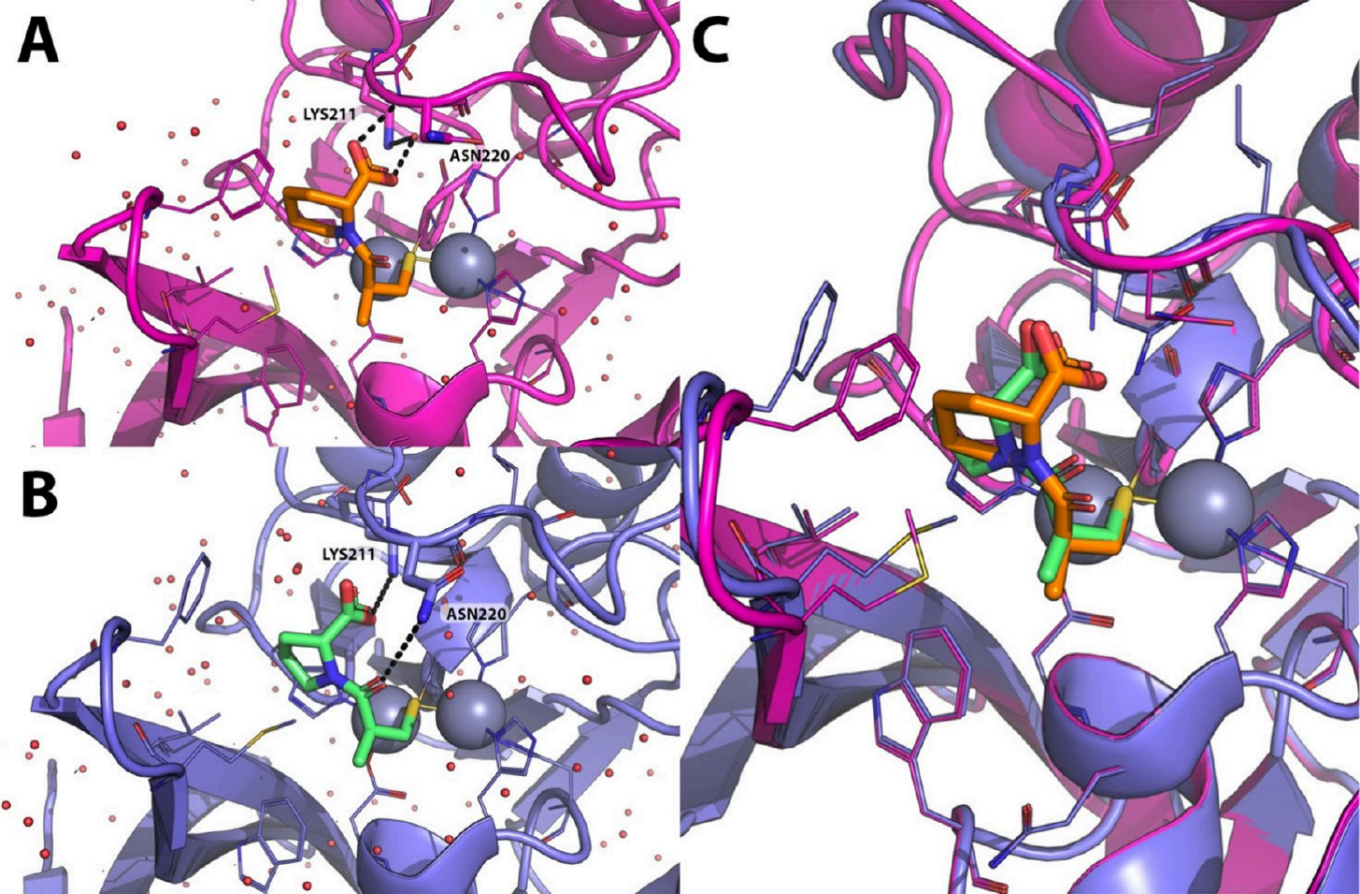
X-ray crystallography structure of NDM-1 in complex with D-captopril and the D-captopril derivative (2*R*)-1-[(2*S*)-2-methyl-3-sulfanyl-propanoyl] piperidine-2-carboxylic acid (wss02122). A) X-ray crystallographic structure of *K. pneumoniae* NMD-1 (magenta cartoon, lines and sticks) in complex with D-captopril derivative wss02122 (orange sticks), PDB-ID: 6LJ0.^58^ B) X-ray crystallographic structure of *K. pneumoniae* NMD-1 (violet cartoon, lines and sticks) in complex with D-captopril (green sticks), PDB-ID: 5ZJ2.^58^ C) Superimposition of the two X-ray crystallography structures. Polar interactions are highlighted by black dashed lines. Zinc ions are represented as gray spheres, while crystallographic water molecules are shown as small red spheres. Residues within 6 Å from the cocrystallized ligands are shown as lines; those involved in binding to the ligands are shown as sticks.

As for the design of siderophore-antibiotic conjugates, selecting a suitable siderophore is also crucial for the development of siderophore-containing MBLi. Bulky natural groups may hinder the drug-target interaction, reducing efficacy. Therefore, simpler artificial siderophores – like monocatechols or 3-hydroxypyridin-4­(1H)-one – are often better suited, as exemplified by cefiderocol. The design of effective linkers also presents a major challenge. Linkers should be easy to synthesize, stable under extracellular conditions and during transport, and capable of releasing the antibiotic at the appropriate site within the bacteria. Cleavable linkers are potentially more practical, although they are less common. Examples include trimethyl lock, disulfide bonds, (acyloxy)­alkyl esters, cephalosporin analogs, and protease-sensitive peptides.^90^

### 6.3 MBLi in Combination with Nanoparticles

In 2023, Gomez et al. reported the promising results in combating antibiotic-resistant bacteria (*Enterococcus sp*. and *Pseudomonas* sp. strains) through the incorporation of MBLi in nanoparticles (silver-, gold-, zinc oxide, and **copper oxide based),** due to their ability to penetrate bacterial cells more efficiently. Incorporating MBLi, such as EDTA, into nanoparticle-based formulations has the potential to enhance the antibacterial activity of traditional antibiotics. In principle, these nanoparticles act as carriers, delivering MBLi directly into the bacterial cells, where they can partially or fully neutralize the activity of MBL enzymes, thus restoring antibiotic activity. Additionally, the nanoparticles themselves possess antimicrobial properties, thus, further improving the effectiveness of the treatment. However, despite these promising results, further studies are needed to assess the compatibility and cytotoxicity of the most effective combinations, ensuring their safety and therapeutic applicability.^83^

## 7 Conclusion and Future Perspectives

The rapid escalation AMR is one of the most pressing global public health challenges. The development of new drugs able to target resistance mechanisms represents a critical step to contrast AMR. In this context, the design and synthesis of novel β-lactamase inhibitors could represent a timely strategy. However, there is currently a lack of approved MBLi on the market. Only a few MBL and dual MBL and SBL inhibitors are undergoing preclinical and clinical trials, including ANT2681 (preclinical phase), taniborbactam (phase III clinical trials), and xeruborbactam (phase I clinical trials). The limited number of available compounds in advanced clinical phases highlights the urgent need of robust medicinal chemistry and drug discovery efforts, including the exploration of drug repurposing and repositioning strategies, to face the AMR issue and speed up the identification of effective compounds targeting resistant bacterial strains. In this context, the introduction of computational methods and big data analysis represents a significant transformation in drug discovery.^91^ Concerning the specific field of MBLi, the relatively high amount of sequence and structural data currently available has triggered some attempts using artificial intelligence (AI) approaches to discover and characterize MBLs and to predict their drug resistance patterns,^92–94^ as well as in the identification of novel MBLi.^95–97^ As data on MBLs and MBLi are rapidly growing, it is expected that AI-based methods, such as machine learning (ML), will become routinely used to accelerate the discovery and optimization of MBLi up to clinical investigations.

Besides, several issues related to limited permeability of MBLi across the bacterial outer membrane still hinder the successful treatment of severe infections caused by multiresistant Gram-negative bacteria. Therefore, future research efforts should prioritize not only the development of novel potent MBLi featuring a broad-spectrum inhibitory profile but also the optimization of their drug-like properties and their ability to successfully penetrate bacterial cells. This can be achieved by harnessing existing strategies, such as the use of siderophores, which have shown promising preliminary results, as well as by developing innovative drug delivery systems to enhance their therapeutic potential. The forthcoming future will definitely see a necessity-driven quick development of this field, where all of the possible approaches herein illustrated, as well as newly disclosed strategies, will be implemented to hit the heart of antibiotic resistance.

## Data Availability

Not applicable.

## ABBREVIATIONS USED

Antimicrobial resistance: (AMR)
metallo-β-lactamases: (MBLs)
serine-β-lactamases: (SBLs)
metallo-β-lactamases inhibitors: (MBLi)
serine-β-lactamases inhibitors: (SBLi)
multidrug resistance: (MDR)
World Health Organization: (WHO)
mobile genetic elements: (MGEs)
resistance nodulation cell divison family: (RND)
ATP binding cassette family: (ABC)
multidrug and toxic compound extrusion family: (MATE)
proteobacterial antimicrobial compound efflux: (PACE)
Imipenemase: (IMP)
Verona integron-encoded metallo-β-lactamase: (VIM)
New Delhi metallo-β-lactamase: (NDM)
extended-spectrum-β-lactamases: (ESBLs)
carbapenem-resistant Enterobacteriaceae: (CRE)
outer membrane vesicles: (OMVs)
Aspergillomarasmine A: (AMA)
imipenem: (IPM)
meropenem: (MEM)
minimum inhibitory concentration: (MIC)
dipicolinic acid: (DPA)
indole-2-carboxylates: (InCs)
high-throughput screening: (HTS)
colloidal bismuth subcitrate: (CBS)
specie: (sp.)
species: (spp.)
urinary tract infection: (UTI)
dihydrobenzo indole: (dBI).
